# Motor and Sensory Deficits in the *teetering* Mice Result from Mutation of the ESCRT Component HGS

**DOI:** 10.1371/journal.pgen.1005290

**Published:** 2015-06-26

**Authors:** Jennifer A. Watson, Bula J. Bhattacharyya, Jada H. Vaden, Julie A. Wilson, Mert Icyuz, Alan D. Howard, Edward Phillips, Tara M. DeSilva, Gene P. Siegal, Andrew J. Bean, Gwendalyn D. King, Scott E. Phillips, Richard J. Miller, Scott M. Wilson

**Affiliations:** 1 Department of Neurobiology, University of Alabama at Birmingham, Evelyn F. McKnight Brain Institute, Civitan International Research Center, Birmingham, Alabama, United States of America; 2 Department of Molecular Pharmacology and Biological Chemistry, Northwestern University, Evanston, Illinois, United States of America; 3 Department of Genetics, University of Alabama at Birmingham, Birmingham, Alabama, United States of America; 4 Department of Physical Medicine and Rehabilitation, University of Alabama at Birmingham, Birmingham, Alabama, United States of America; 5 Departments of Pathology, Surgery and Cell, Developmental and Integrative Biology, University of Alabama at Birmingham, Birmingham, Alabama, United States of America; 6 Department of Neurobiology and Anatomy and Graduate School of Biomedical Sciences, The University of Texas Health Science Center at Houston, Houston, Texas, United States of America; 7 Division of Pediatrics, The University of Texas M.D. Anderson Cancer Center, Houston, Texas, United States of America; University of California San Diego, UNITED STATES

## Abstract

Neurons are particularly vulnerable to perturbations in endo-lysosomal transport, as several neurological disorders are caused by a primary deficit in this pathway. In this report, we used positional cloning to show that the spontaneously occurring neurological mutation *teetering* (*tn*) is a single nucleotide substitution in hepatocyte growth factor-regulated tyrosine kinase substrate (*Hgs/Hrs*), a component of the endosomal sorting complex required for transport (ESCRT). The *tn* mice exhibit hypokenesis, muscle weakness, reduced muscle size and early perinatal lethality by 5-weeks of age. Although HGS has been suggested to be essential for the sorting of ubiquitinated membrane proteins to the lysosome, there were no alterations in receptor tyrosine kinase levels in the central nervous system, and only a modest decrease in tropomyosin receptor kinase B (TrkB) in the sciatic nerves of the *tn* mice. Instead, loss of HGS resulted in structural alterations at the neuromuscular junction (NMJ), including swellings and ultra-terminal sprouting at motor axon terminals and an increase in the number of endosomes and multivesicular bodies. These structural changes were accompanied by a reduction in spontaneous and evoked release of acetylcholine, indicating a deficit in neurotransmitter release at the NMJ. These deficits in synaptic transmission were associated with elevated levels of ubiquitinated proteins in the synaptosome fraction. In addition to the deficits in neuronal function, mutation of *Hgs* resulted in both hypermyelinated and dysmyelinated axons in the *tn* mice, which supports a growing body of evidence that ESCRTs are required for proper myelination of peripheral nerves. Our results indicate that HGS has multiple roles in the nervous system and demonstrate a previously unanticipated requirement for ESCRTs in the maintenance of synaptic transmission.

## Author Summary

Endocytic trafficking involves the internalization, endosomal sorting and lysosomal degradation of cell surface cargo. Many factors involved in endosomal sorting in mammalian cells have been identified, and mutations in these components are associated with a variety of neurological disorders. While the function of endosomal sorting components has been intensely studied in immortalized cell lines, it is not known what role these factors play in endosomal sorting in the nervous system. In this study, we show that the *teetering* (*tn*) gene encodes the *h* *epatocyte* *g* *rowth factor regulated tyrosine kinase* *s* *ubstrate* (*Hgs*), a core component of the endosomal sorting pathway. The *tn* mice exhibit several signs of motor neuron disease, including reduced muscle mass, muscle weakness and motor abnormalities. Although HGS is predicted to be required for the lysosomal degradation of receptor tyrosine kinases, there was no change in the levels of receptor tyrosine kinases in the spinal cords of the *tn* mice. Instead, we found that HGS is required for synaptic transmission at the neuromuscular junction and for the proper myelination of the peripheral nervous system.

## Introduction

In neurons, endosomal transport and sorting of internalized cargo affects the abundance of plasma membrane proteins and regulates a diverse group of cellular processes such as signal transduction and synaptic vesicle recycling [1–3]. A group of evolutionarily conserved cytosolic proteins referred to as the endosomal sorting complexes required for transport (ESCRT) associate with the endosomal membrane and direct the sorting of internalized cargo [4,5]. Four distinct ESCRT complexes, ESCRT-0, -I, -II and—III, act sequentially to contribute to the sorting of membrane proteins, thus determining if endocytosed receptors are recycled back to the cell surface or are further sorted into intraluminal vesicles (ILVs) within specialized endosomes called multivesicular bodies (MVBs) for their eventual degradation by lysosomes [6]. Receptor degradation rates affect the duration of receptor signaling, and alterations in the turnover of activated receptors have been implicated in a variety of disease processes [7–10].

The ESCRT-0 complex, composed of HGS and signal transducing adaptor molecule 1 (STAM1) [11–13], is essential for the initial recognition of ubiquitinated cargo that will be sorted at the endosomal membrane and degraded upon endo-lysosomal fusion [14,15]. HGS is believed to serve as a master regulator of endo-lysosomal trafficking by binding ubiquitinated cargo and initiating the recruitment of the ESCRT-I, -II, and—III components [16–19]. Given its role in the early stages of endosomal sorting of internalized cell surface receptors, it has been suggested that HGS also plays an important role in the recycling of cargo to the plasma membrane [20–22]. In addition, HGS can bind synaptosomal associated protein 25 (SNAP-25) and prevent SNARE complex formation, thereby inhibiting neurotransmitter secretion [23] and endosomal fusion [24]. Studies in *Drosophila* have also implicated HGS in synaptic protein homeostasis and synaptic vesicle rejuvenation [25]. However, despite the insights into HGS function gained from the studies described above, the precise role of HGS in endosomal trafficking has not been determined in the mammalian nervous system.

Disruption of endosomal transport is associated with many neurological disorders [26]. In some instances, a direct link has been demonstrated between the genetic defect and endosomal dysfunction. For example, mutations in both ESCRT components and endosomal-associated factors have been attributed to neurodegeneration [26]. Mutations in multivesicular body protein 2B (CHMP2B), a component of the ESCRT-III complex, are linked to both frontotemporal dementia and amyotrophic lateral sclerosis [7,27,28]. In addition, mutations in kinesin family member 5A (KIF5A) and SPARTIN, which facilitate endosomal trafficking, are associated with motor neuron dysfunction in hereditary spastic paraplegia [29–31]. Together, these findings suggest that motor neurons are particularly vulnerable to deficits in endosomal trafficking and that genes in this pathway may be excellent candidate genes for inherited motor neuron diseases.

In addition to its association with neurodegeneration in the central nervous system, ESCRT dysfunction is also associated with peripheral demyelinating diseases. Mutations in lipopolysaccharide-induced tumor necrosis factor-alpha factor (LITAF)/SIMPLE, that interacts with HGS and STAM1 in the ESCRT-0 complex [32,33], cause a demyelinating form of Charcot Marie Tooth disease [34,35], suggesting that ESCRT-0 is required for myelin formation or stability. By directing activated receptor tyrosine kinases to the lysosome, ESCRT-mediated endosomal sorting provides a mechanism to prevent aberrant signaling that is believed to result in demyelination and degeneration of peripheral axons.

In this study, we used a positional cloning approach to demonstrate that the neurological phenotypes observed in the *tn* mice are due to a point mutation in *Hgs* that leads to hypomorphic expression of HGS. The subsequent reduction of HGS caused motor and sensory deficits that were accompanied by peripheral nerve hypermyelination and dysmyelination. Further analysis of motor function demonstrated that the *tn* mice had reduced muscle development and both structural and functional alterations at the NMJ. These deficits in neuromuscular function were associated with the accumulation of ubiquitinated proteins at the synapse. This report represents the first demonstration that a mutation in an ESCRT-0 component causes a motor and sensory neuropathy and suggests that HGS is required for the sorting of ubiquitinated proteins at the synapse to maintain synaptic transmission at the NMJ.

## Results

### Cloning of the *tn* mutation

**Fig 1:**
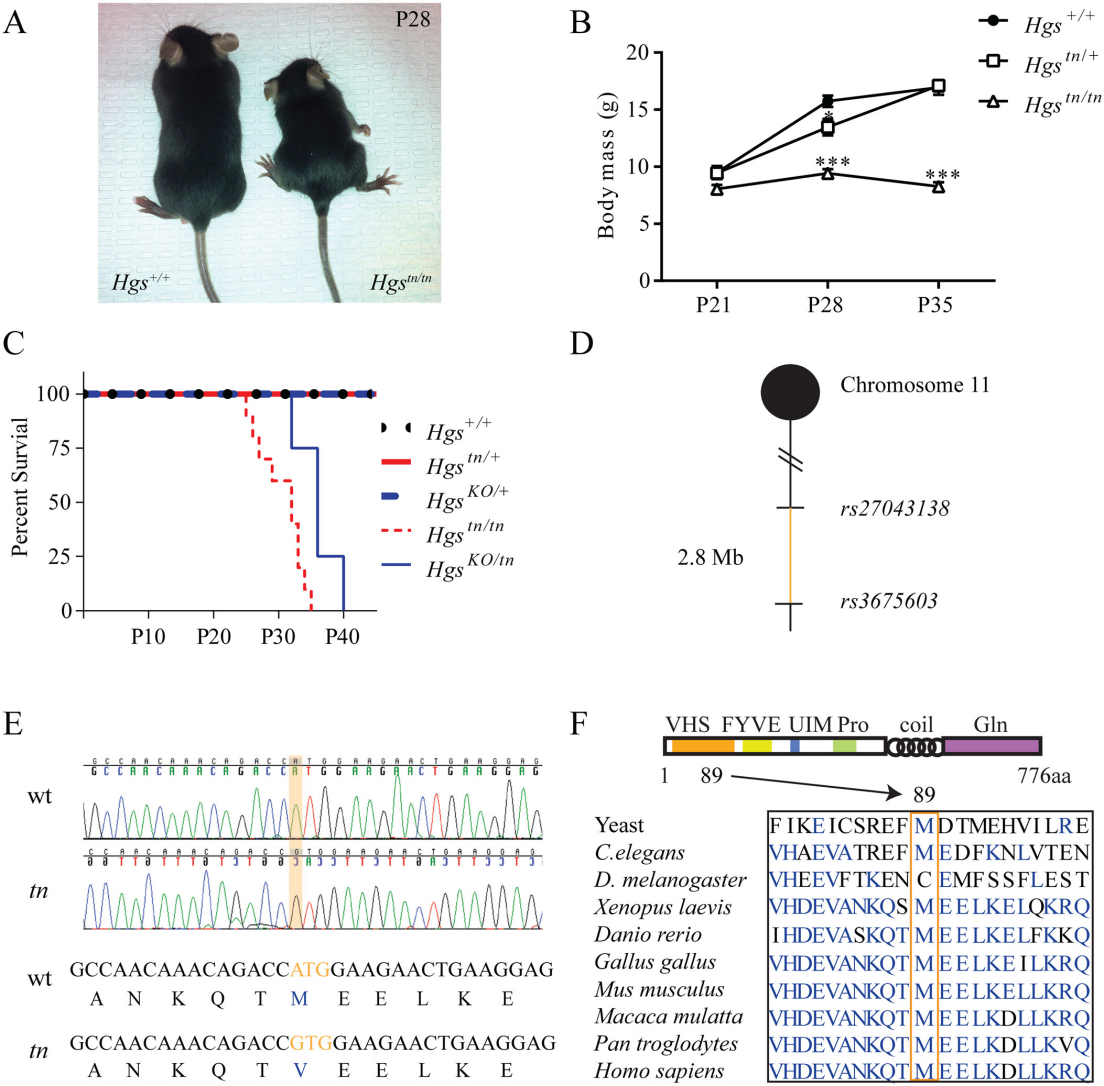
Positional cloning and phenotypic analysis of the *tn* mice. (A) Image showing reduced size of 4-week-old *Hgs* ^*tn/tn*^ mice relative to wild type *HGS* ^*+/+*^ mice. (B) Body mass of 3- to 5-week-old *HGS* ^*+/+*^, heterozygous *Hgs* ^*tn/+*^ and *Hgs* ^*tn/tn*^ mice. n > 6 mice per genotype. A two-way anova was used to find a significant effect of genotype on body mass. Symbols represent unpaired t-tests corrected for multiple comparisons using the Holm-Sidak method (C) Kaplan-Meier survival curve of wild type (*Hgs* ^*+/+*^) and *Hgs-*mutant mice. The *Hgs* ^*KO*^ allele does not complement the *Hgs* ^*tn*^ allele. A Mantel-Cox test with p<0.001 demonstrated a significant difference between the survival curves of the *Hgs* ^*tn/tn*^ and *Hrs* ^*KO/tn*^ mice as compared to the *Hg* ^*KO/+*^ *Hgs* ^*tn/+*^, and *Hgs* ^*+/+*^ mice. (D) Meiotic linkage map depicting SNP markers that define the *tn* critical region. (E) Genomic sequencing of *HGS* revealed an adenine to guanine change in the *Hgs* ^*tn/tn*^ mice, resulting in a methionine to valine substitution at amino acid 89 of HGS. (F) Schematic of HGS protein structure in eukaryotes, demonstrating the conservation of the methionine residue at position 89 in the VHS domain (orange box). Data are shown as mean ± SE. *p < 0.05 and ***p < 0.001.

The *tn* mutation arose spontaneously in a C3H/HeJ inbred mouse line, resulting in a progressive neurodevelopmental disorder that first presents at 3 weeks of age [36]. Homozygous *tn* mice exhibit reduced growth, ataxia, hypokinesis and premature death at 4 to 5 weeks of age (Fig 1A–1C) [36]. These deficits are thought to be due to dysgenesis of the brainstem and spinal cord [36]. To identify the minimal chromosomal region harboring the *tn* mutation, a congenic mouse line was generated by backcrossing the *tn* mutation onto the C57BL/6J genetic background. Analysis of single nucleotide polymorphisms localized the *tn* mutation to a 2.8 Mb region on distal chromosome 11 (Fig 1D). We then performed transcriptome analysis of brain RNA to identify nucleotide changes in the coding sequences of the genes located within the *tn* critical region. Sequence analysis of the 87 genes within the *tn* critical region only revealed a single nucleotide substitution in the *Hgs* gene. Genomic sequencing of the *Hgs* gene from the *tn* mice confirmed an adenine to guanine transition at position 265, which resulted in a methionine to valine substitution at amino acid 89 (M89V) (Fig 1E). The methionine residue at position 89 of HGS is highly conserved throughout eukaryotic phylogeny (Fig 1F).

The M89V substitution is located in the N-terminal Vps27-HGS-STAM (VHS) domain of HGS that is involved in substrate binding [37,38]. To confirm that this mutation in HGS is responsible for the *tn* phenotype, a genetic complementation assay was performed by breeding mice that were heterozygous for the *tn* allele (*Hgs* ^*tn/+*^) to mice that were heterozygous for a knockout allele of *Hgs* (*Hgs* ^*tm1S/+*^, henceforth referred to as *Hgs* ^*KO/+*^) [39]. The resulting *Hgs* ^*KO/tn*^ offspring exhibited perinatal lethality by postnatal day 40 (Fig 1C) and a neurological phenotype similar to the *tn* mice, confirming that the *tn* gene was allelic to *Hgs*. Since the *Hgs* ^*KO/KO*^ mice die by embryonic day 11 [39], the enhanced survival of the *Hgs* ^*KO/tn*^ mice indicated that the *tn* allele was not a null allele of *Hgs*.

### HGS expression in the nervous system is developmentally regulated

**Fig 2:**
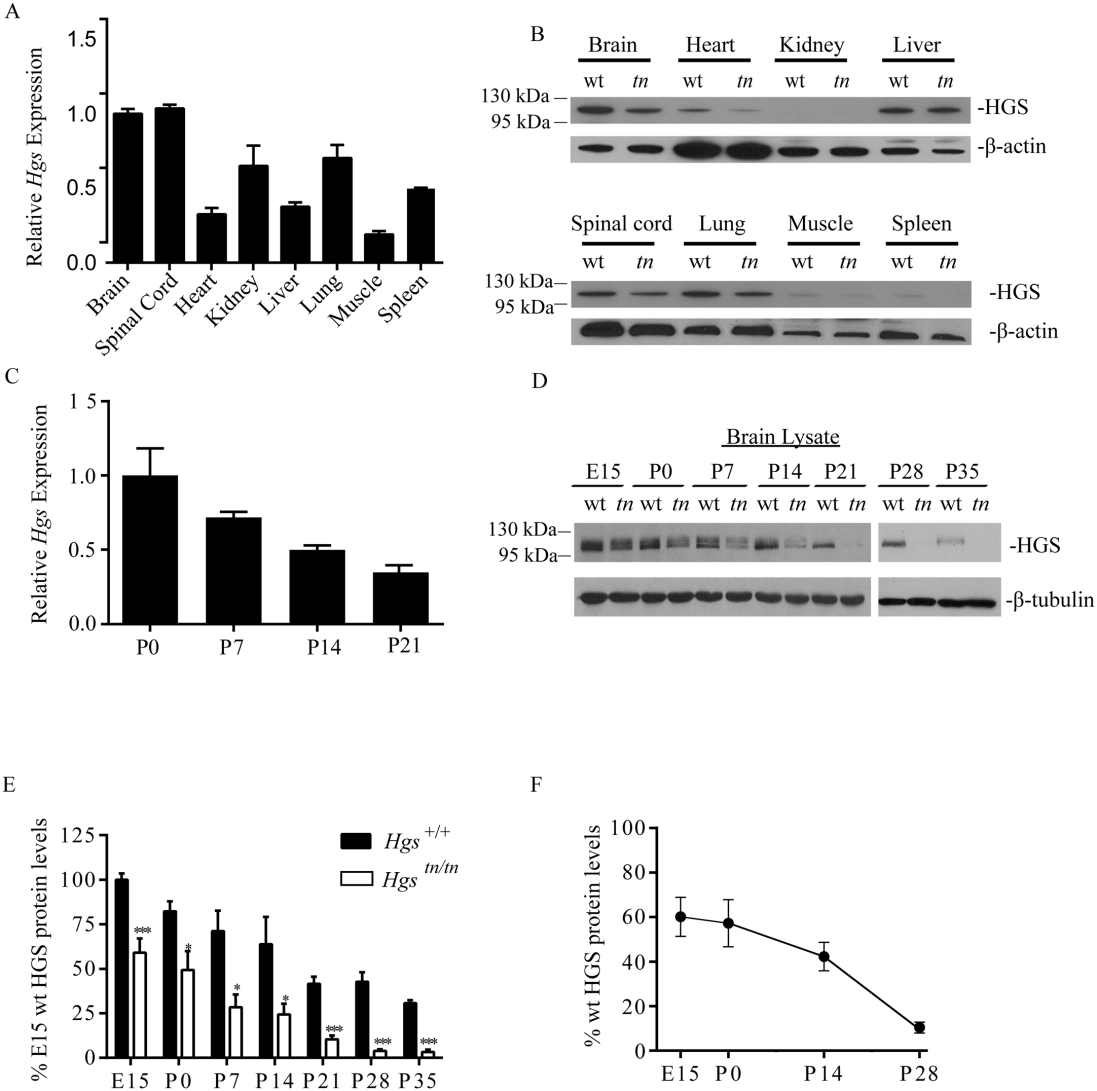
HGS expression in *Hgs* ^*+/+*^ and *Hgs* ^*tn/tn*^ tissues. (A) qPCR analysis of *Hgs* mRNA expression in 4-week-old *Hgs* ^*+/+*^ tissues. Transcript level is expressed relative to *Hgs* level found in the brain. (B) Representative immunoblot of HGS expression in 4-week-old *Hgs* ^*+/+*^ (wt) and *Hgs* ^*tn/tn*^ (*tn*) mice. β-actin was used as a loading control. (C) qPCR analysis of *Hgs* levels from the brains of *Hgs* ^*+/+*^ mice during postnatal development. (D) Representative immunoblot analysis of HGS expression from embryonic day 15 (E15) to postnatal day 35 (P35) in *Hgs* ^*+/+*^ (wt) and *Hgs* ^*tn/tn*^ (*tn*) brain lysates. β-tubulin is used as a loading control. (E) Quantitation of developmental time course of HGS expression in *Hgs* ^*+/+*^ (wt) and *Hgs* ^*tn/tn*^ (*tn*) mice expressed as percent of E15 *Hgs* ^*+/+*^ levels. Symbols represent unpaired t-tests corrected for multiple comparisons using the Holm-Sidak method. A one way anova with a Geisser-Greenhouse adjustment demonstrated a significant difference between time points. (F) Quantitation of HGS expression in *Hgs* ^*tn/tn*^ mice expressed as a percent of *Hgs* ^*+/+*^ controls at each developmental time point. Data are shown as ± SE. Symbols represent unpaired t-tests. *p<0.05 and ***p<0.001.

To investigate the distribution of *Hgs* expression *in vivo*, we performed quantitative polymerase chain reaction (qPCR)on reverse-transcribed RNA isolated from 4-week-old *Hgs* ^*+/+*^ wild type mice. *Hgs* was expressed in all tissues examined, with the highest levels of *Hgs* detected in the brain and spinal cord (Fig 2A). Consistent with the qPCR data, immunoblot analysis also revealed high levels of HGS in the nervous system of wild type mice (Fig 2B). While the *tn* mutation resulted in a significant reduction of HGS in the nervous system of the *Hgs* ^*tn/tn*^ mice, it differentially affected HGS levels in non-neuronal tissues (Fig 2B). Examination of the heart, liver, spleen, kidney, adrenal gland, thalamus, testis, and ovaries from the *Hgs* ^*tn/tn*^ mice revealed a striking absence of pathology in these tissues, suggesting that HGS provides an essential function that is unique to the nervous system.

Because *Hgs* ^*tn/tn*^ mice display a progressive neurological disease starting around 3 weeks of age, it was important to establish a developmental profile of *Hgs* expression in the brain. We found that *Hgs* levels undergo a normal down regulation in the *Hgs* ^*+/+*^ mice during early postnatal development (Fig 2C). Immunoblot analysis also showed the highest levels of HGS during embryonic development, with abundance decreasing throughout postnatal development (Fig 2D and 2E). The M89V mutation led to reduced expression of HGS at all time points examined (Fig 2D–2F), with the most severe loss of HGS occurring during the time when the neurological phenotypes were most pronounced in the *Hgs* ^*tn/tn*^ mice. In addition to the primary immunoreactive band that migrated at approximately 110 kDa, a second higher molecular weight band migrating at approximately 120 kDa, which may represent a post-translationally modified form of HGS [40–42], was also detected early in development.

### Absence of cell death in the hippocampus of *Hgs* ^*tn/tn*^ mice

**Fig 3:**
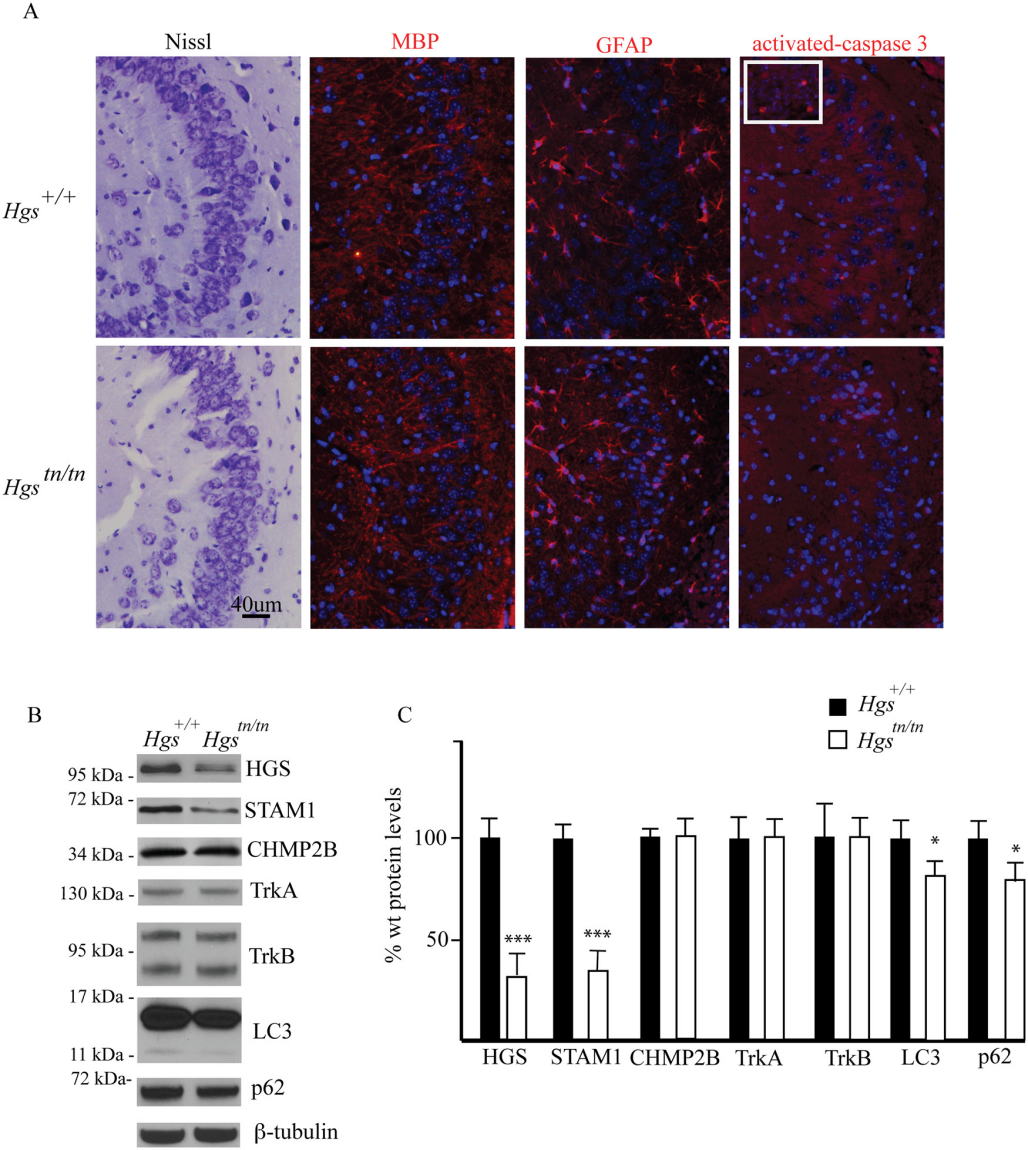
Comparison of hippocampal structure and protein expression between *Hgs* ^*tn/tn*^ and *Hgs* ^*+/+*^ mice. (A) CA3 hippocampal sections from 4-week-old *Hgs* ^*+/+*^ and *Hgs* ^*tn/tn*^ mice were stained for Nissl, myelin basic protein (MBP), glial fibrillary protein (GFAP) and activated caspase 3. Inset is positive control (E13 embryo) for activated caspase-3. Nuclei are stained with DAPI (blue). (B) Representative immunoblot of hippocampal lysates from 4-week-old *Hgs* ^*+/+*^ and *Hgs* ^*tn/tn*^ mice. Blots were probed for the ESCRT components HGS, STAM1, and CHMP2B, the receptor tyrosine kinases TrkA and TrkB, and the autophagic markers LC3 and p62. (C) Quantitation of immunoblots from hippocampal lysates. n = 3 mice per genotype. Data are shown as mean ± SE. *p<0.05 and ***p<0.001.

A previous study of a neuronal-specific knockout of *Hgs* demonstrated multiple deficits in the hippocampus that included increased ubiquitin staining of CA3 pyramidal cells, increased CA3 pyramidal cell death, and reduced CA3 pyramidal cell numbers [43]. To determine whether the *Hgs* ^*tn/tn*^ mice exhibited any alterations in the number of CA3 pyramidal cells or an increase in cell death, we measured the number of CA3 cells and looked for evidence of increased cell death by performing Nissl and activated caspase-3 staining, respectively, in the hippocampi of wild type and *Hgs* ^*tn/tn*^ mice. Unlike what was reported for the neuronal-specific *Hgs* knockout mice, we did not detect any change in the number of CA3 pyramidal cells or an increase in cell death markers in the *Hgs* ^*tn/tn*^ hippocampus (Fig 3A). In addition, the levels of hippocampal myelin basic protein were similar in the *Hgs* ^*tn/tn*^ and *Hgs* ^*+/+*^ mice. Moreover, while increased glial fibrillary acid protein (GFAP) abundance is often associated with neurodegeneration, there was no increase in GFAP immunoreactivity in the hippocampus of the *Hgs* ^*tn/tn*^ mice compared to wild type controls (Fig 3A). Similar to what we observed in total brain extracts, there was a significant reduction in both HGS and STAM1 levels in the hippocampus of the *Hgs* ^*tn/tn*^ mice. Since mutations in CHMP2B are associated with neurodegeneration in humans, we examined the effect of loss of HGS on CHMP2B levels and found no detectable differences in the hippocampus between the *Hgs* ^*tn/tn*^ or wild type controls. Examination of the levels of the receptor tyrosine kinases TrkA and TrkB, which are putative substrates for HGS, in hippocampal extracts from the *Hgs* ^*tn/tn*^ and *Hgs* ^*+/+*^ mice also did not reveal any significant differences in expression (Fig 3B and 3C).

As a component of the endosome, HGS has been implicated in the maturation of autophagosomes, as the loss of HGS expression results in increased levels of markers of autophagy in mammalian cell lines [44]. To investigate if HGS is required for autophagy in neurons, we examined the expression of the autophagosome marker microtubule-associated protein 1A/1B-light chain 3 (LC3) and the autophagy substrate p62 in hippocampal lysates from wild type and *Hgs* ^*tn/tn*^ mice. In contrast to the increased levels of LC3 and p62 that are observed when autophagy is impaired [44], we detected a reduction in the levels of both LC3 and p62 in the hippocampus of the *Hgs* ^*tn/tn*^ mice as compared to controls (Fig 3B and 3C). Together, these data indicate that loss of HGS does not appear to result in increased cell death or a significant impairment of autophagy in the hippocampus of the *Hgs* ^*tn/tn*^ mice.

### *Hgs* ^*tn/tn*^ mice exhibit motor and sensory deficits

**Fig 4:**
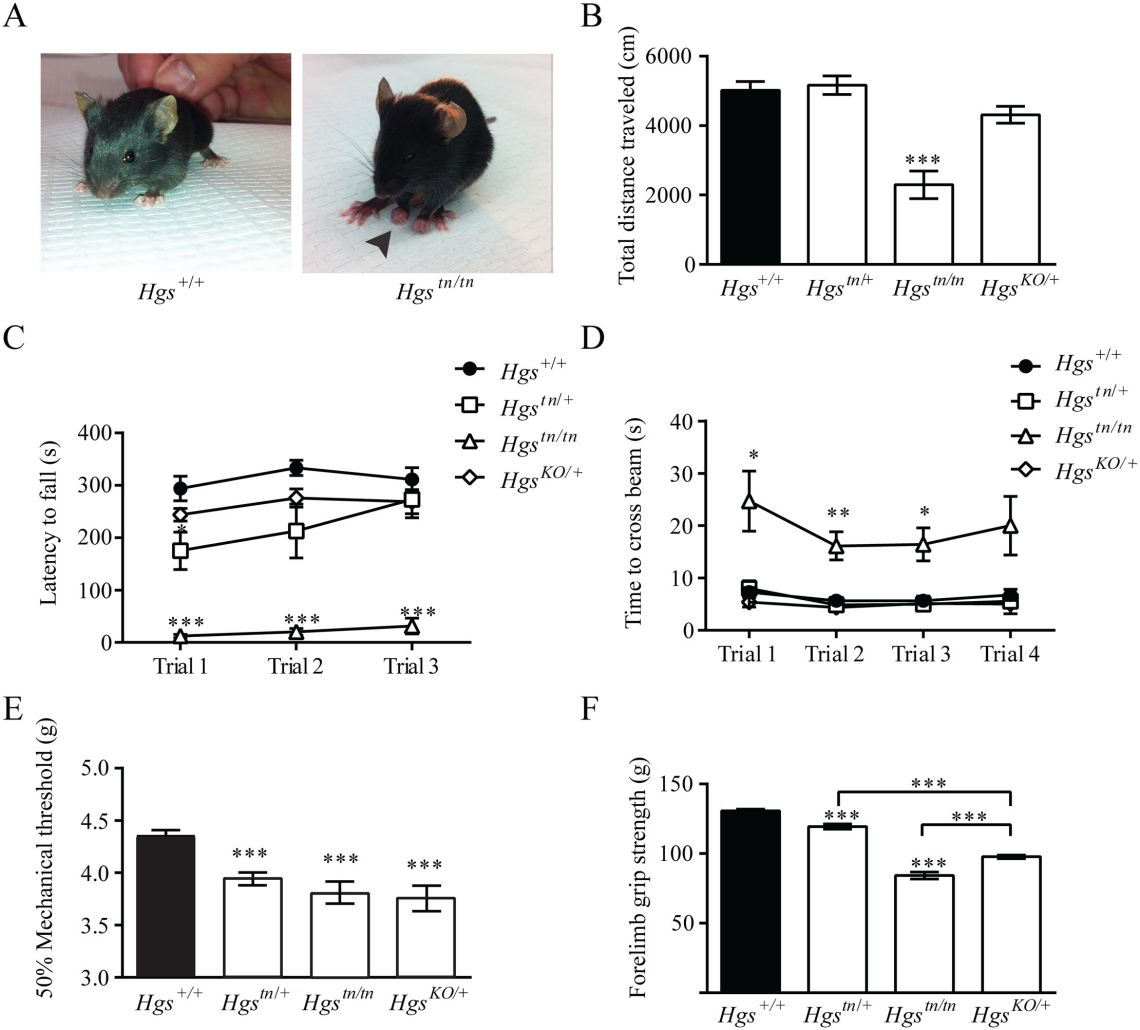
Gene dosage effects of HGS expression on motor and sensory function in 3- to 4-week-old mice. (A) Reduced HGS expression results in clawed paws in *Hgs* ^*tn/tn*^ mice. Behavioral assays of (B) open field, (C) rotarod, (D) elevated beam, (E) von Frey and (F) forelimb grip strength for *Hgs* ^*+/+*^ (black), *Hgs* ^*tn/+*^, *Hgs* ^*tn/tn*^ and *Hgs* ^KO/+^ mice. n = 6 mice per genotype for all assays except rotarod, where n = 4. Symbols represent unpaired t-tests corrected for multiple comparisons using the Holm-Sidak method. Data are shown as mean ± SE and n>6 animals per genotype for all assays except rotarod, where n = 4. A two-way anova demonstrated a significant effect of genotype on rotarod and elevated beam performance. Data are shown as mean ± SE. *p<0.05, **p<0.01 and ***p<0.001.

Several neurological diseases are caused by mutations that lie within genes involved in the endosomal sorting of membrane proteins [45–48]. To examine whether HGS deficiency resulted in motor and sensory deficits commonly seen in patients with inherited neuropathies, we performed a series of behavioral assays on our mutant *Hgs* mouse lines. By 3 weeks of age, the *Hgs* ^*tn/tn*^ mice exhibited significant motor deficits as demonstrated by the presence of clawed paws, decreased locomotion in an open field assay, impaired rotarod performance and an increased time to transverse an elevated beam compared to controls (Fig 4A–4D). In addition, the *Hgs* ^*tn/tn*^ mice also demonstrated increased tactile sensitivity that was consistent with a heightened pain response when examined using the von Frey assay (Fig 4E) and had reduced forelimb muscle strength as compared to age-matched controls (Fig 4F).

The initial description of the *tn* mice indicated that the heterozygous *tn* mice displayed motor abnormalities in a prolonged swimming assay [36]. We therefore investigated whether heterozygous *Hgs* ^tn/+^ and *Hgs* ^*KO/+*^ mice also exhibited motor and sensory abnormalities in our assays. Both the *Hgs* ^*KO/+*^ mice and the *Hgs* ^tn/+^ mice displayed increased pain sensitivity and reduced muscle strength (Fig 4E and 4F), indicating that the *tn* mutation is a loss-of-function mutation and that a 50% loss of HGS is sufficient to cause peripheral nervous system dysfunction.

### *Hgs* ^*tn/tn*^ mice display hypermyelination, dysmyelination, myelin infoldings, and increased axonal caliber

**Fig 5:**
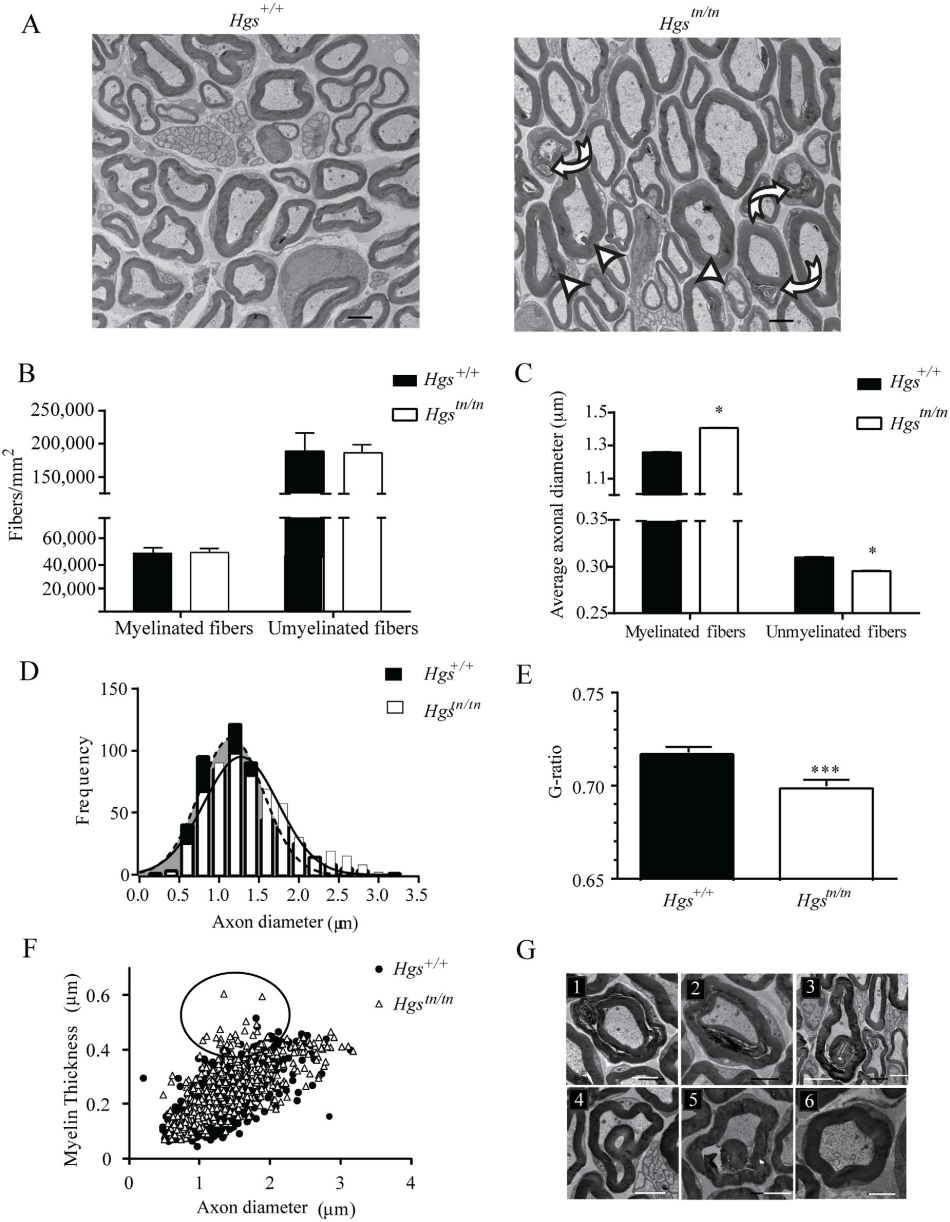
Examination of sciatic nerves from 4-week-old *Hgs* ^*+/+*^ and *Hgs* ^*tn/tn*^ mice. (A) Electron micrograph of sciatic nerves from 4-week-old *Hgs* ^*tn/tn*^ and *Hgs* ^*+/+*^ mice. Scale bar, 2 μm. Arrowheads indicate hypermyelinated fibers, curved arrows indicated disorganized myelin and arrows indicate demyelination. (B) Quantitation of axon density in myelinated and unmyelinated nerves. (C) Quantitation of average myelinated and unmyelinated axon diameters. (D) Histogram of frequency of axon diameters demonstrating an increase in large diameter myelinated axons in the sciatic nerves of *Hgs* ^*tn/tn*^ mice relative to *Hgs* ^*+/+*^ controls. Shaded region represents axonal size distribution from *Hgs* ^*+/+*^ mice. An unpaired t-test with a Welch’s correction demonstrated a significant difference in the distribution of axonal size frequency between *Hgs* ^+/+^ and *Hgs* ^tn/tn^ mice. (E) Quantitation of the ratio of axon diameter to total fiber thickness (G-ratio). Symbols represent unpaired t-tests. (F) Relationship between myelin thickness and axon diameter in *Hgs* ^*+/+*^ and *Hgs* ^*tn/tn*^ sciatic nerves. Circled region depicts 1.0–2.0 μm diameter axons that are affected in the *Hgs* ^*tn/tn*^ sciatic nerves. (G) Representative micrographs of myelin pathology in *Hgs* ^*tn/tn*^ nerves demonstrating (1–2) Tomaculous fibers, (3–5) myelin infoldings compared to (6) *Hgs* ^*+/+*^controls. n = 3 mice per genotype. Scale bar, 5 μm. Data are shown as mean ± SE. *p<0.05 and ***p<0.001.

Alterations in axon number or peripheral nerve myelination are common features of inherited peripheral neuropathies and are thought to be important contributors to motor and sensory dysfunction. To examine whether loss of HGS expression results in axonal loss, demyelination, or dysmyelination that could contribute to the behavioral deficits in the *Hgs* ^*tn/tn*^ mice, we compared the sciatic nerves of 4-week-old *Hgs* ^+/+^ and *Hgs* ^*tn/tn*^ mice by transmission electron microscopy. Although no change in the density of myelinated and unmyelinated axons was found in the *Hgs* ^*tn/tn*^ mice (Fig 5A and 5B), there was a significant increase in the diameter of myelinated axons and a significant decrease in the diameter of unmyelinated axons in the 4-week-old *Hgs* ^*tn/tn*^ mice as compared to controls (Fig 5C). This increase in the average axonal diameter of myelinated fibers was attributed to a shift in distribution towards a greater number of large diameter axons in the *Hgs* ^*tn/tn*^ mice (Fig 5D). Morphometric analysis of myelin structure also revealed a small but significant decrease in the G-ratio (the ratio of the inner axonal diameter to the total fiber diameter) of sciatic nerves from the *Hgs* ^*tn/tn*^ mice (Fig 5E). This decrease was attributed to a 22% increase in the myelin thickness of small-diameter axons that are between 1.0 to 2.0 μm in diameter (Fig 5F, circled region).

Several alterations in myelin structure were also observed in the sciatic nerves of the *Hgs* ^*tn/tn*^ mice (Fig 5G). These alterations included the presence of tomacula, a prominent thickening of compact myelin with redundant loops, which likely contributed to the decreased G-ratio observed in the *Hgs* ^*tn/tn*^ mice. Additionally, many regions of the myelin sheaths were disorganized in the *Hgs* ^*tn/tn*^ mice (Fig 5G1–5G2) and contained structural alterations such as myelin infoldings (Fig 5G3–5G5) as compared to controls (Fig 5G6).

**Fig 6:**
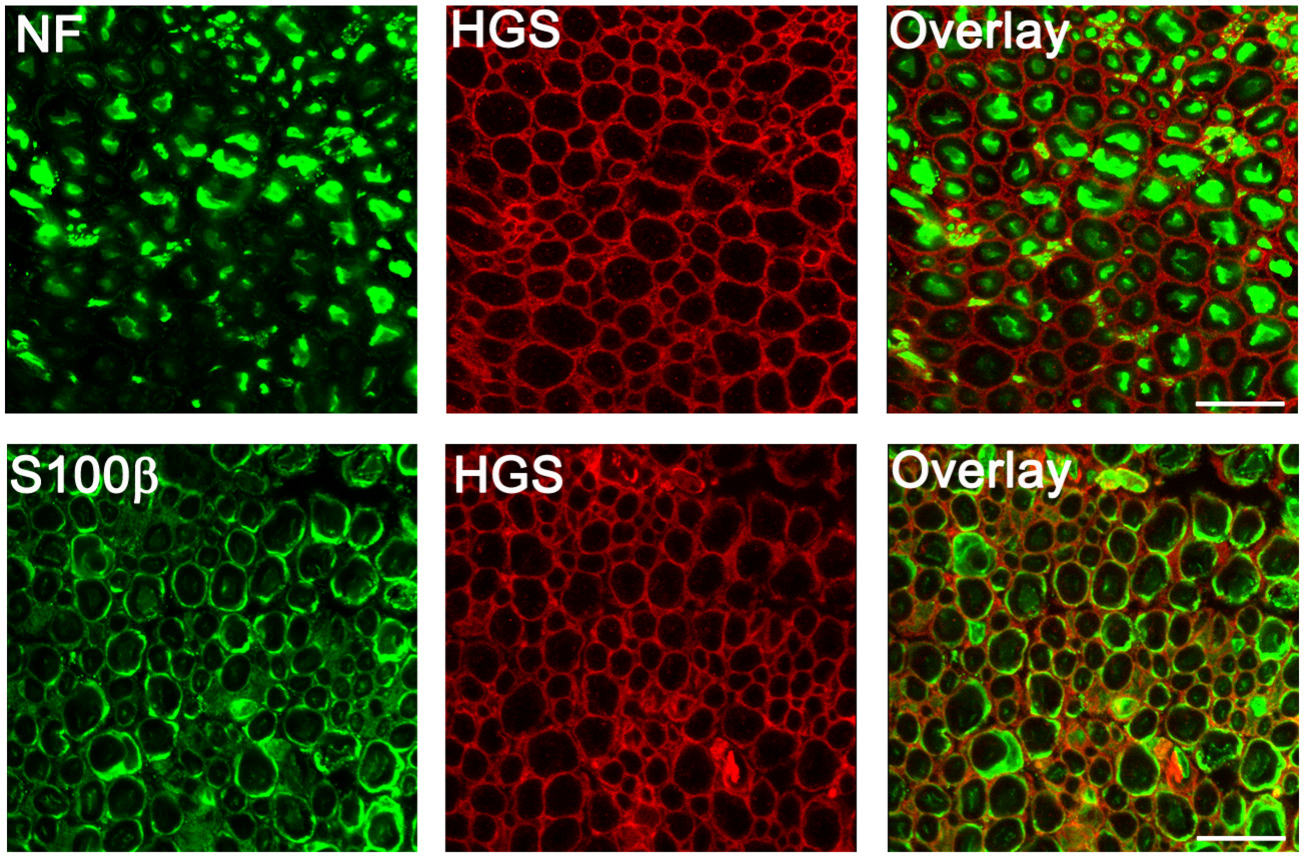
Distribution of HGS in sciatic nerves of 4-week-old *Hgs* ^*+/+*^mice. Top panel, cross sections of sciatic nerves stained with antibodies against neurofilament (NF, green) and HGS (red). Bottom panel, sciatic nerves were stained with antibodies to the Schwann cell marker S100β (green) and HGS (red). Scale bar, 10 μm.

The changes in myelination that were observed in the *Hgs* ^*tn/tn*^ mice could be attributed to loss of HGS in either axons or Schwann cells. However, when we examined HGS expression in the sciatic nerves of wild type mice by indirect immunofluorescence (Fig 6), we found that HGS staining did not overlap with the neurofilament staining in sciatic nerve axons. Rather, when Schwann cells were visualized with antibodies to the cytoplasmic protein S100β, HGS localized to the external boundary of the Schwann cell body. These findings are in agreement with a previous study detecting HGS transcripts in Schwann cells [49].

### ESCRT and RTK expression in sciatic nerves of *Hgs* ^*tn/tn*^ mice

**Fig 7:**
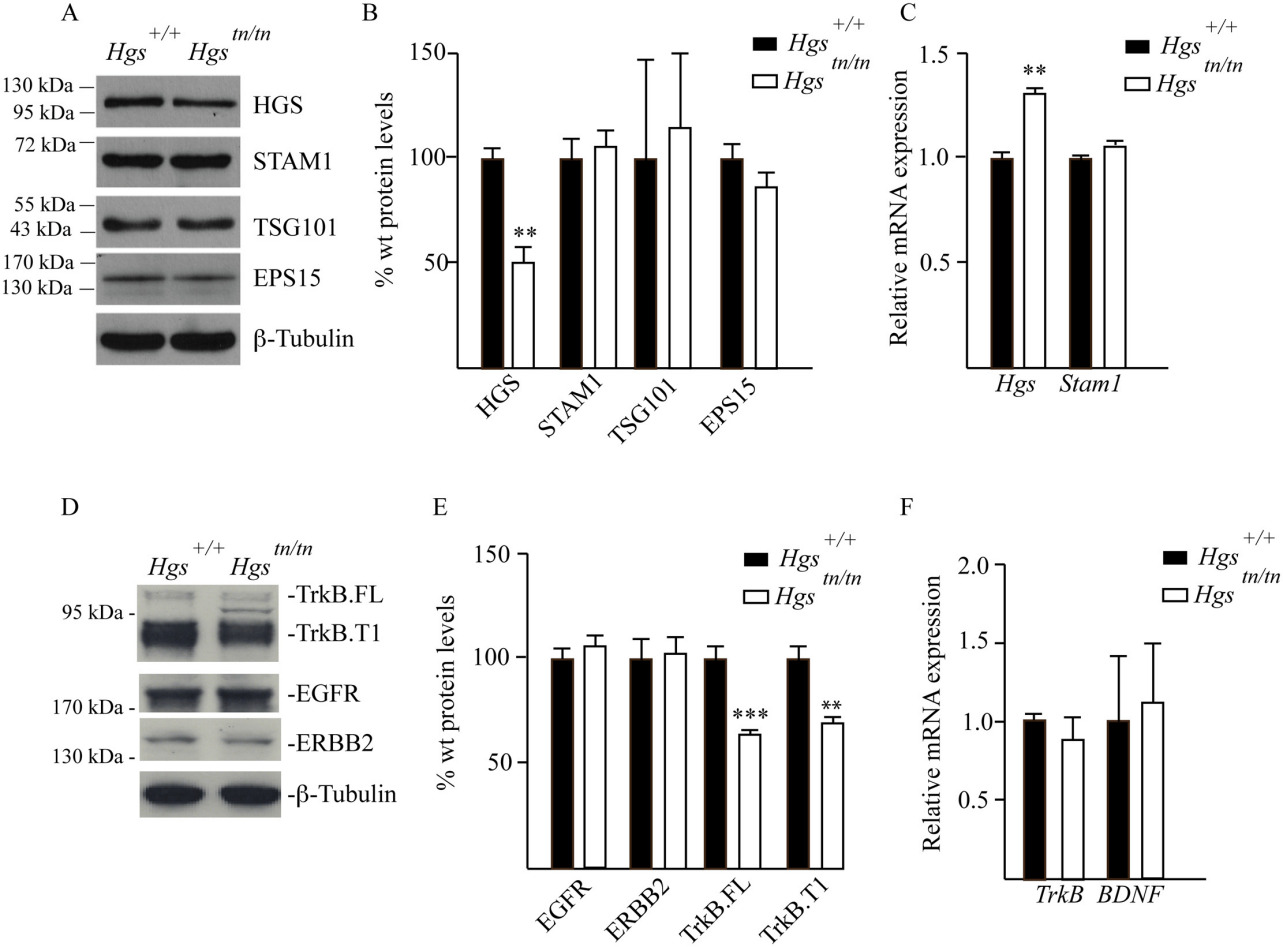
Analysis of ESCRT and RTK expression in the sciatic nerves of 4-week-old *Hgs* ^*+/+*^ and *Hgs* ^*tn/tn*^ mice. (A) Representative immunoblot of ESCRT expression in sciatic nerve extracts. β-tubulin was used as a loading control. (B) Quantitation of immunoblots of ESCRT expression in sciatic nerve extracts. (C) qPCR analysis of ESCRT-0 components in the sciatic nerve. (D) Representative immunoblot of TrkB.FL and TrkB.T1, EGFR and ERBB2 in sciatic nerves. β-tubulin was used as a loading control. (E) Quantitation of receptor tyrosine kinases in the sciatic nerve. (F) qPCR analysis of *TrkB* and *BDNF* in the sciatic nerve. Symbols represent unpaired t-tests corrected for multiple comparisons using the Holm-Sidak method. Data are shown as mean ± SE. n > 3 mice per genotype. **p<0.01 and ***p<0.001.

In cell culture models, the stability of STAM1 appears to be dependent upon HGS expression in a transcript-independent manner [16,50]. However, this relationship between ESCRT-0 components has not been investigated in the nervous system. We therefore examined the effect of the *tn* mutation on the expression of ESCRT components and their putative substrates in the sciatic nerves of the *Hgs* ^*tn/tn*^ mice. While the HGS levels were reduced by 50% in the sciatic nerves of 4-week-old *Hgs* ^*tn/tn*^ mice compared to controls, there were no significant differences in the levels of STAM1, tumor susceptibility protein 101 (TSG101) or epidermal growth factor receptor substrate 15 (EPS15) in the sciatic nerve extracts from the *Hgs* ^*tn/tn*^ and *Hgs* ^*+/+*^ mice (Fig 7A–7C). Analysis of receptor tyrosine kinases (RTKs) sorted by the ESCRT pathway showed that loss of HGS resulted in a significant reduction in both the full length (TrkB.FL) and truncated (TrkB.T1) isoforms of TrkB in the sciatic nerves of the *Hgs* ^*tn/tn*^ mice that was not associated with a corresponding reduction in *TrkB* mRNA levels (Fig 7D–7F). In contrast, reduction of HGS did not affect the level of either the epidermal-growth factor receptor (EGFR) or the receptor tyrosine-protein kinase erbB-2 (ERBB2) (Fig 7D and 7E).

### *Hgs* ^*tn/tn*^ mice have reduced STAM1 expression in the spinal cord but do not exhibit motor neuron loss or altered levels of RTKs

**Fig 8:**
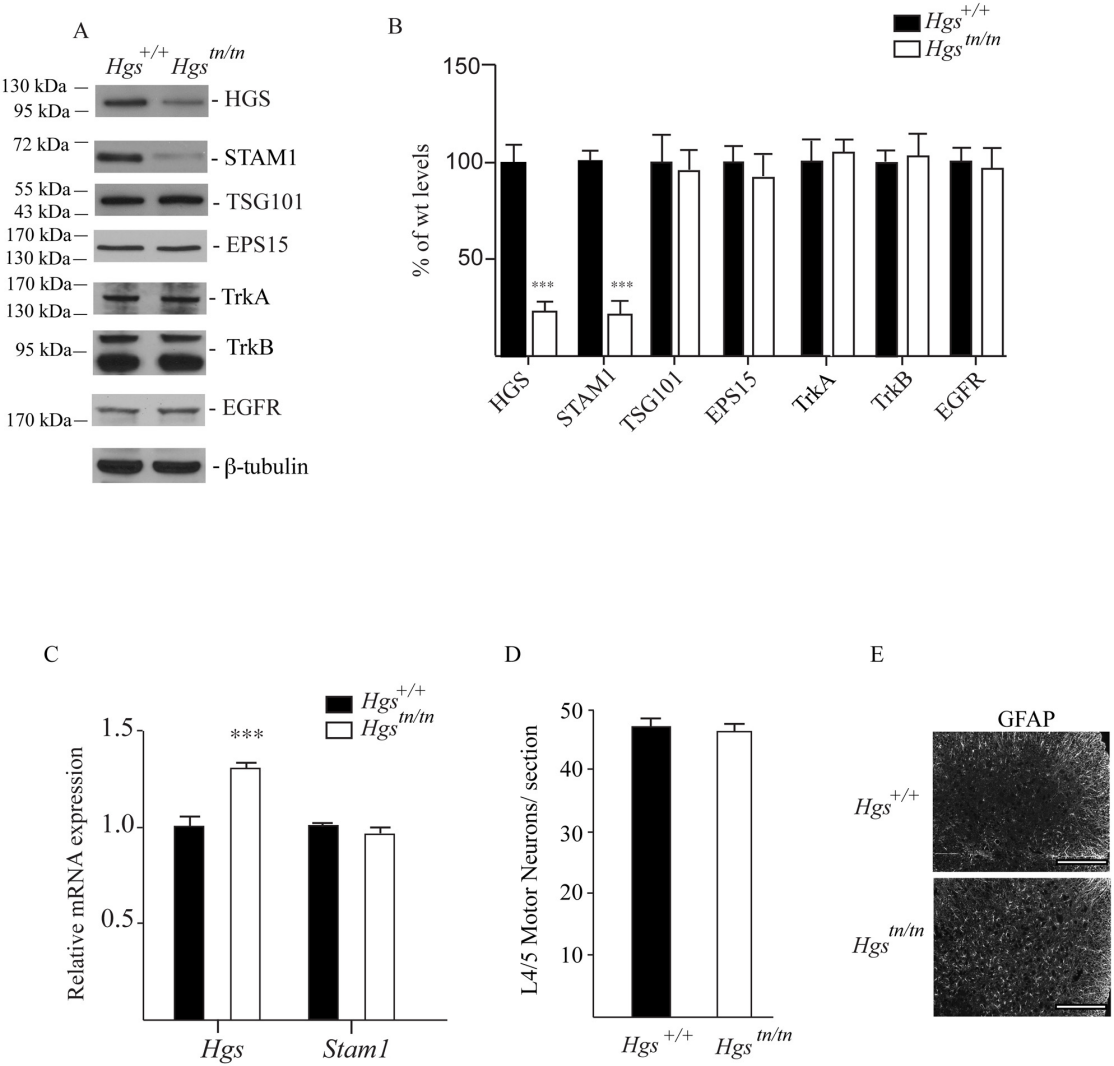
Levels of HGS-interacting proteins and putative substrates in spinal cord extracts of 4-week-old *Hgs* ^*+/+*^ and *Hgs* ^*tn/tn*^ mice. (A) Representative immunoblot and (B) quantitation of the ESCRT-0 proteins HGS and STAM1, the ESCRT-I protein TSG101, the ESCRT-0 interacting proteins EPS15, and the receptor tyrosine kinases TrkB, TrkA and EGFR in the spinal cords of *Hgs* ^*+/+*^ and *Hgs* ^*tn/tn*^ mice. β-tubulin was used as a loading control. (C) qPCR analysis of *Hgs* and *Stam1* in the spinal cords of *Hgs* ^*+/+*^ and *Hgs* ^*tn/tn*^ mice. Levels are expressed relative to levels found in wild type *Hgs* ^*+/+*^ mice. Symbols represent unpaired t-tests corrected for multiple comparisons using the Holm-Sidak method. Data are shown as mean ± SE. (D) Motor neuron counts from lumbar segments 4/5 from *Hgs* ^*+/+*^ and *Hgs* ^*tn/tn*^ mice. n = 3 mice per genotype. (E) Immunostaining of *Hgs* ^*+/+*^ and *Hgs* ^*tn/tn*^ L4/5 segments with GFAP. Scale bar, 100 μm. Data are shown as mean ± SE. n > 3 mice per genotype. ***p<0.001.

Since loss of HGS resulted in a dramatic motor phenotype in the *Hgs* ^*tn/tn*^ mice, but had only a modest effect on peripheral nerve myelination, we examined the effect of reduced HGS expression on the abundance of ESCRT-0 components in the spinal cords of 4-week-old mice (Fig 8A and 8B). Despite the significant increase in *Hgs* transcript levels in the *Hgs* ^*tn/tn*^ mice (Fig 8C), HGS levels were reduced 70% in the spinal cords of the *Hgs* ^*tn/tn*^ mice compared to controls (Fig 8A and 8B). Similarly, we also observed an 80% reduction in the levels of STAM1 in the spinal cords of the *Hgs* ^*tn/tn*^ mice with no corresponding decrease in *Stam1* transcript abundance (Fig 8A–8C). When we examined whether the loss of HGS affected the abundance of other proteins reported to interact with HGS [51–53], there was no difference in the levels of EPS15 or TSG101 in the spinal cords of the *Hgs* ^*tn/tn*^ and *Hgs* ^*+/+*^ mice (Fig 8A and 8B). Although HGS appears to influence the abundance of some receptor tyrosine kinases in immortalized cell lines [22,53–58] and TrkB in the sciatic nerves (Fig 7), the levels of EGFR, TrkA and TrkB found in the spinal cords of the *Hgs* ^*tn/tn*^ mice were similar to those observed in the *Hgs* ^*+/+*^ control mice (Fig 8A and 8B).

To determine if the movement disorder in the *Hgs* ^*tn/tn*^ mice was associated with a loss of motor neurons, we compared motor neuron numbers in the ventral horn of 4- to 5-week-old *Hgs* ^*tn/tn*^ and *Hgs* ^*+/+*^ mice. Consistent with the axonal density studies on the sciatic nerves, there was no significant difference in the number of motor neurons (Fig 8D) in the lumbar 4/5 region between the *Hgs* ^*tn/tn*^ and *Hgs* ^*+/+*^ mice. However, loss of HGS in the *Hgs* ^*tn/tn*^ mice did result in increased GFAP staining in the lumbar 4/5 region of the spinal cord (Fig 8E).

### Reduced muscle growth and increased acetylcholine receptor expression in *Hgs* ^*tn/tn*^ mice

**Fig 9:**
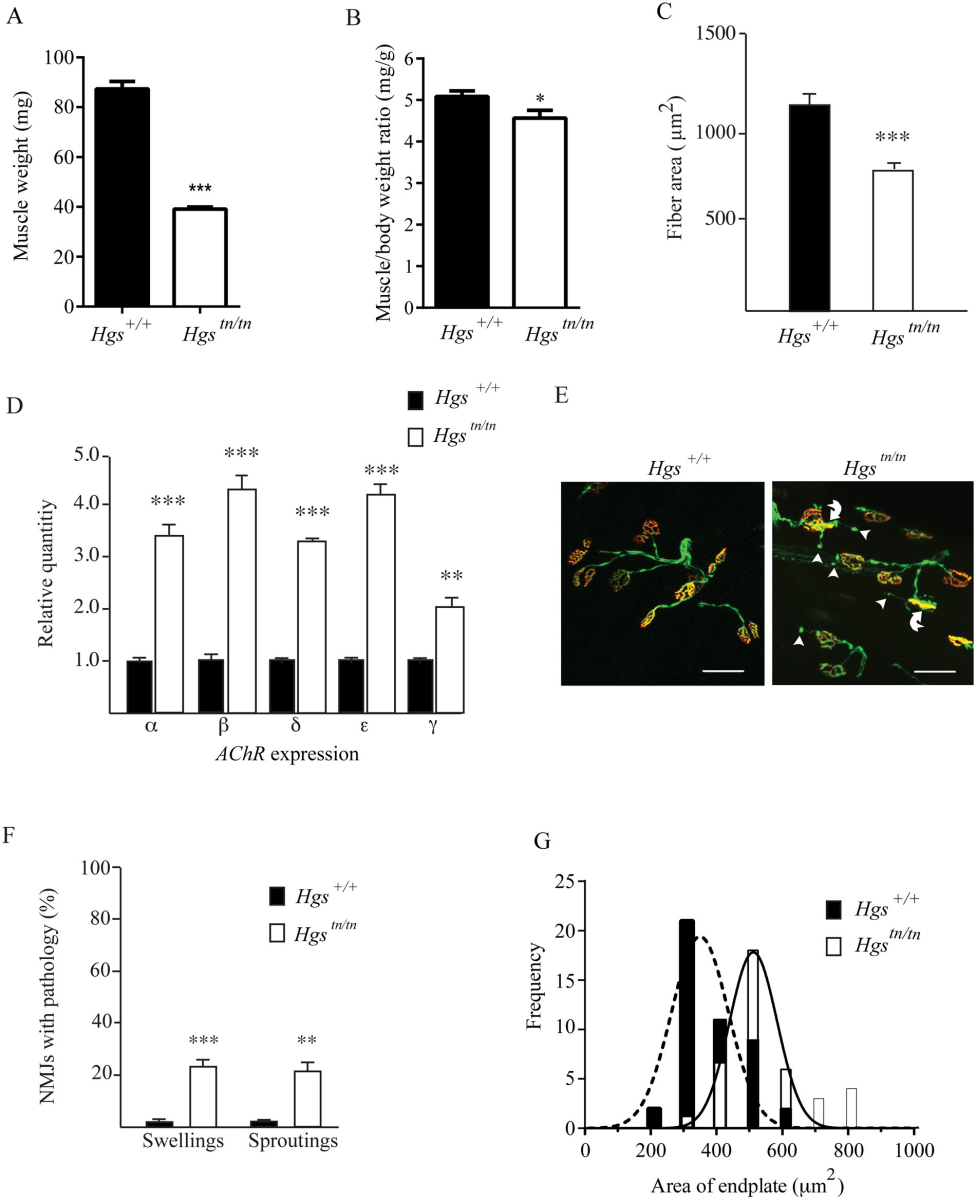
Alterations in muscles and motor endplates in the *Hgs* ^*tn/tn*^ mice. (A) Wet weights of gastrocnemius muscles from 4 week-old *Hgs* ^*+/+*^ and *Hgs* ^*tn/tn*^ mice. (B) Ratio of gastrocnemius muscle weights to body mass. n > 6 mice per genotype for each time point. (C) Gastrocnemius muscle fiber size measurements for *Hgs* ^*+/+*^ and *Hgs* ^*tn/tn*^ mice. n > 6 mice per genotype. Symbols represent unpaired t-tests. (D) qPCR analysis of *AChR-α*, *AChR-β*, *AChR-δ*, *AChR-ε*, and *AChR-γ* mRNAs from the gastrocnemius muscles of 4-week-old *Hgs* ^*+/+*^ and *Hgs* ^*tn/tn*^ mice. n **>** 3 mice per genotype. Symbols represent unpaired t-tests corrected for multiple comparisons using the Holm-Sidak method. (E) Motor endplate pathology in the *Hgs* ^*tn/tn*^ mice. TA muscle fibers from *Hgs* ^*+/+*^ and *Hgs* ^*tn/tn*^ mice containing the *Thy1*-*Yfp* transgene (green) were stained with TRITC-α-bungarotoxin (red) to label the postsynaptic receptors. The presynaptic axons and nerve terminals are shown in green. Arrowheads mark ultra-terminal sprouting, and curved arrows mark swollen presynaptic terminals. Scale bar, 20 μm. (F) Quantitation of terminal swellings and terminal sprouting from *Hgs* ^*+/+*^ and *Hgs* ^*tn/tn*^ mice. n > 6 mice per genotype. Symbols represent unpaired t-tests corrected for multiple comparisons using the Holm-Sidak method. (G) Histogram of endplate area defined by TRITC-α-bungarotoxin (red) labeling of the postsynaptic AChR in *Hgs* ^*+/+*^ and *Hgs* ^*tn/tn*^ mice. An unpaired t-test with a Welch’s correction demonstrated a significant difference in the distribution of endplate size frequency between *Hgs* ^+/+^ and *Hgs* ^tn/tn^ mice. n > 6 mice per genotype. Data are shown as mean ± SE. *p<0.05, **p<0.01 and ***p<0.001.

The *Hgs* ^*tn/tn*^ mice exhibit several signs of neuromuscular disease, including muscle weakness and decreased motor performance. When we compared the muscle mass between the *Hgs* ^*tn/tn*^ mice and the *Hgs* ^*+/+*^ controls, we detected a significant difference in gastrocnemius weights at 4 weeks of age (Fig 9A and 9B). While we did not detect angular muscle fibers or centrally located nuclei in the muscle sections of the *Hgs* ^*tn/tn*^ mice, which are common features of motor neuron disease, there was a 32% reduction in muscle fiber size in the *Hgs* ^*tn/tn*^ mice (Fig 9C). These changes were consistent with decreased motor neuron input onto the muscle fibers. Since acetylcholine receptor (AChR) abundance is inversely correlated with motor neuron input onto muscle fibers, we performed qPCR on gastrocnemius muscle RNA isolated from 4-week-old wild type and *Hgs* ^*tn/tn*^ mice. Both the embryonic and adult acetylcholine receptor subunit mRNAs were significantly increased in the gastrocnemius muscles of the *Hgs* ^*tn/tn*^ mice (Fig 9D).

### Loss of HGS results in structural and functional changes at the NMJ

Alterations in motor endplate structure are found in several models of neuromuscular disease [59–62]. Using mice that express yellow fluorescent protein (YFP) in the motor axons to visualize motor neuron terminals from tibialis anterior (TA) muscles, we found that every postsynaptic AChR cluster in the wild type mice was innervated by a motor neuron axon (Fig 9E). Although all of the AChR clusters from the 4-week-old *Hgs* ^*tn/tn*^ mice were also innervated, we observed a significant increase in the number of terminals that exhibited either terminal swellings or sproutings (Fig 9F), which are phenotypes that are often observed in mice with motor neuron disease [59,62,63]. In addition, there was a significant increase in endplate size in the *Hgs* ^*tn/tn*^ mice (Fig 9G), which resembles the increase in endplate area observed in mice lacking the vesicular acetylcholine transporter [61]. These results are consistent with the idea that reduced HGS expression results in decreased neurotransmitter release onto *Hgs* ^*tn/tn*^ muscles.

**Fig 10:**
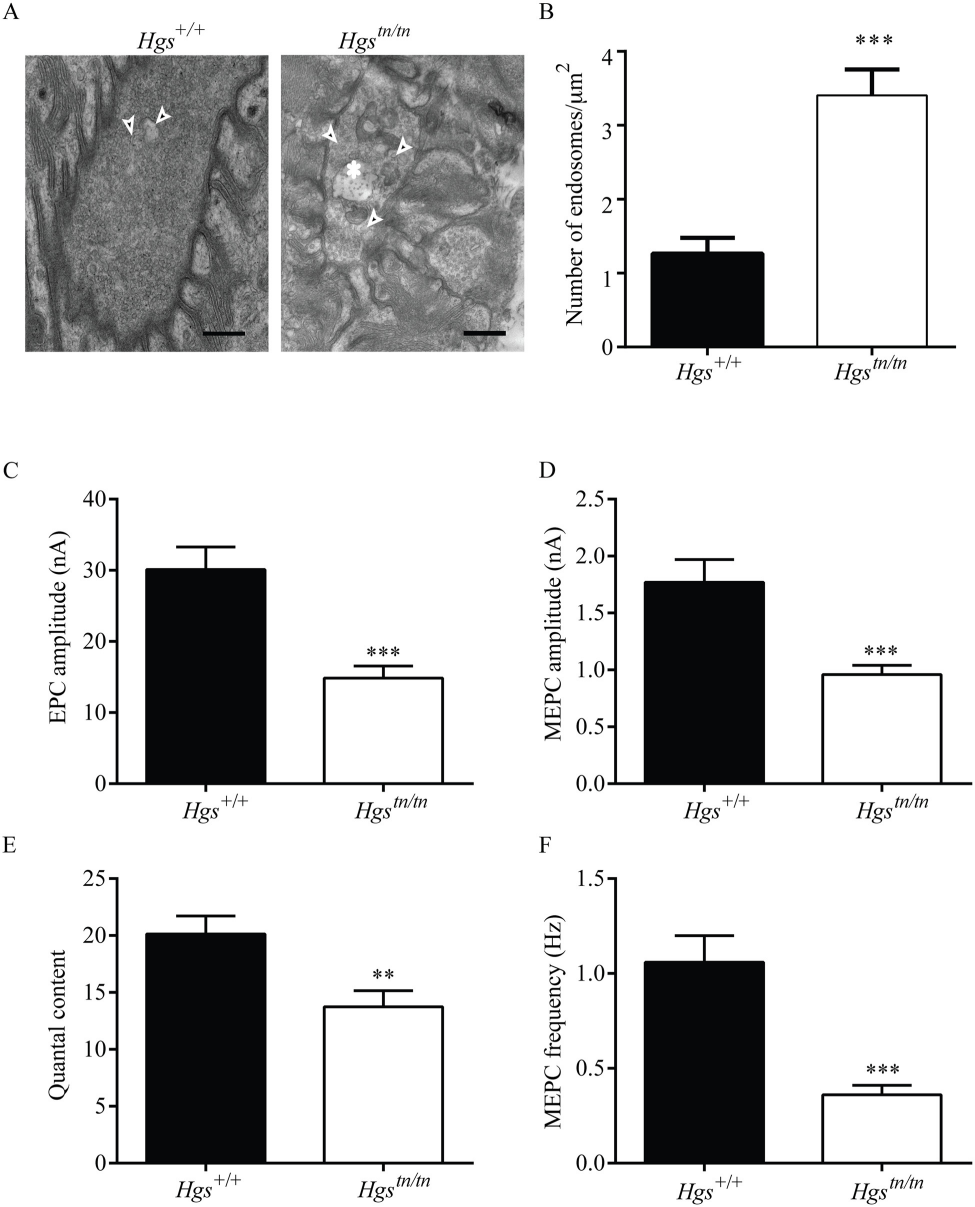
Loss of HGS increases the number of endosome-like structures and results in synaptic transmission deficits at the NMJ. (A) Representative electron micrographs of NMJs in the TA muscle from 4-week-old *Hgs* ^*+/+*^ and *Hgs* ^*tn/tn*^ mice. Arrowheads point to endosomes-like structures. Asterisk marks an MVB in the *Hgs* ^*tn/tn*^ presynaptic terminal. No MVBs were observed in *Hgs* ^*+/+*^ terminals. Scale bar, 500 μm. (B) Quantitation of endosome-like structures at the motor axon terminals. Symbol represents unpaired t-tests. (C) A 50% reduction in EPC amplitudes was observed in the endplates from 3-week-old *Hgs* ^*tn/tn*^ mice (n = 12 endplates from 6 mice) as compared to controls (n = 12 endplates from 5 mice). (D) MEPC amplitudes were reduced in the *Hgs* ^*tn/tn*^ mice (n = 19 endplates from 6 mice) to 46% of *Hgs* ^*+/+*^ controls (n = 15 endplates from 8 mice). (E) Quantal content was significantly lower in *Hgs* ^*tn/tn*^ mice (n = 8 endplates from 6 mice) than in *Hgs* ^*+/+*^ controls (n = 12 endplates from 5 mice). (F) Reduced HGS expression results in a 65% reduction in MEPC frequency at the TA muscles of *Hgs* ^*tn/tn*^ mice (n = 17 endplates from 6 mice) compared to *Hgs* ^*+/+*^controls (n = 14 endplates from 5 mice). Symbol represents unpaired t-tests **p<0.01 and ***p<0.001.

By sorting internalized membrane proteins from the endosome to the lysosome, the ESCRT complexes are thought to regulate the turnover of internalized cargo [26]. To investigate whether loss of HGS altered endosome or MVB abundance at motor neuron terminals, we compared the NMJs from wild type and *Hgs* ^*tn/tn*^ TA muscles by electron microscopy (Fig 10A). Loss of HGS expression resulted in an increase in the number of endosomal-like structures in the *Hgs* ^*tn/tn*^ mice (Fig 10A and 10B). While we did not observe any MVBs in the electron micrographs taken from wild type NMJs, we observed several MVBs (0.23 MVBs/μm^2^) in the motor neuron terminals of the *Hgs* ^*tn/tn*^ mice. No significant difference was observed in the number of autophagosomes in the NMJs of the *Hgs* ^*tn/tn*^ and *Hgs* ^*+/+*^mice.

HGS is thought to have a role in regulating exocytosis through its interactions with components of presynaptic nerve terminals [23,52]. To test whether HGS is required to maintain neurotransmitter release, we used two-electrode voltage-clamp analysis to study the release of acetylcholine at the NMJs of 3-week-old *Hgs* ^*tn/tn*^ and *Hgs* ^*+/+*^ mice and observed a pronounced effect of HGS reduction on neurotransmitter release in the TA muscles of the *Hgs* ^*tn/tn*^ mice (Fig 10C). The amplitude of evoked neurotransmitter release, measured as endplate currents (EPCs) and obtained by stimulating sciatic nerves, was decreased by 50% in the *Hgs* ^*tn/tn*^ mice as compared to controls (Fig 10C). The EPC amplitude is determined by both the number of synaptic vesicles released after nerve stimulation (quantal content) and the amplitude of the muscle response to the neurotransmitter released from a single vesicle (quantal amplitude) [60,64]. The amplitude of miniature endplate currents (MEPCs), representing a response to a single quanta or vesicle, was also reduced in the *Hgs* ^*tn/tn*^ mice compared to controls (Fig 10D). This finding suggests that there was either a decrease in the amount of neurotransmitter per vesicle or that there was a reduction in the sensitivity or abundance of post-synaptic receptors in the *Hgs* ^*tn/tn*^ mice. However, the increase in AChR expression observed in the *Hgs* ^*tn/tn*^ mice compared to controls, and the similarites between the phenotypes of the *Hgs* ^*tn/tn*^ mice and mice lacking the vesicular ACh transporter, both suggest that there was a reduction in the level of acetylcholine found in the synaptic vesicles of the *Hgs* ^*tn/tn*^ mice. The finding of decreased MEPC amplitudes in the *Hgs* ^*tn/tn*^ mice also suggests that HGS may be required post-synaptically for stable AChR expression.

In addition, a reduction in quantal content (the number of vesicles released by evoked stimulation), which is calculated by dividing EPC amplitude by MEPC amplitude, was also observed in the *Hgs* ^*tn/tn*^ mice compared to controls (Fig 10E). To further examine the effect of HGS loss on presynaptic function, we measured the frequency of spontaneous neurotransmitter release and determined that there was a 66% decrease in MEPC frequency in the *Hgs* ^*tn/tn*^ mice compared to controls (Fig 10F). These findings indicate that loss of HGS severely affects synaptic transmission by reducing acetylcholine release at the NMJ and that these changes in presynaptic function likely contribute to the motor deficits seen in the *Hgs* ^*tn/tn*^ mice.

### Loss of HGS results in the accumulation of ubiquitinated proteins at the synapse

**Fig 11:**
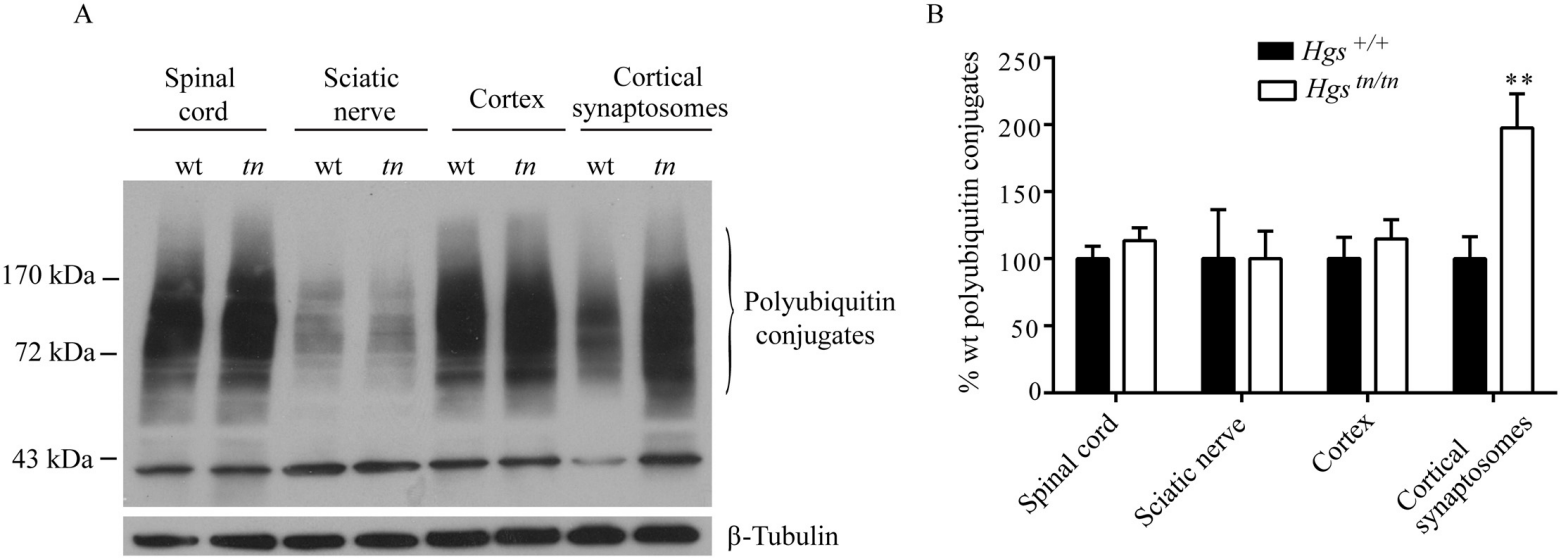
Effect of reduced HGS expression on ubiquitin conjugates in the nervous system of *Hgs* ^*tn/tn*^ mice. (A) Representative immunoblot of ubiquitin conjugates from the nervous system of 4-week-old *Hgs* ^*+/+*^ (wt) and *Hgs* ^*tn/tn*^ (*tn*) mice. (B) Quantitation of immunoblots. n = 3 per genotype. Symbols represent unpaired t-tests corrected for multiple comparisons using the Holm-Sidak method. Data are shown as mean ± SE. **p<0.001.

HGS is involved in the sorting of ubiquitinated proteins, and our studies indicate that loss of HGS has a profound effect on synaptic transmission at the NMJ. To investigate whether HGS is required for the sorting of ubiquitinated proteins at the synapse, we examined the level of ubiquitinated proteins in both the total and synaptosomal fractions prepared from cerebral cortices of wild type and *Hgs* ^*tn/tn*^ mice. While the level of ubiquitin conjugates in the spinal cord, sciatic nerve and cortex were similar between the *Hgs* ^*tn/tn*^ mice and controls, we observed a 2-fold increase in the level of ubiquitinated proteins isolated from the cortical synaptosomes of the *Hgs* ^*tn/tn*^ mice (Fig 11A and 11B). This compartment-specific effect of HGS reduction on the levels of ubiquitin conjugates suggests that HGS is involved in the sorting of ubiquitinated proteins at the synapse and that proper endosomal sorting at the NMJ is required to maintain synaptic transmission.

## Discussion

These studies demonstrate that the neurological deficits observed in the *tn* mice are caused by a hypomorphic mutation in the ESCRT-0 component *Hgs* which significantly reduced HGS levels in the central nervous system. By 4 weeks of age, the *Hgs* ^*tn/tn*^ mice show significant defects in motor and sensory function as well as reduced muscle development, increased muscle AChR expression, terminal sproutings and swellings of motor axons, and increased numbers of endosomes and MVBs at the motor neuron axon terminals. In addition to these structural changes in the motor axon terminals, we also observed a significant reduction in both spontaneous and evoked vesicular release of acetylcholine at the NMJs of the *Hgs* ^*tn/tn*^ mice, suggesting that HGS-dependent sorting of proteins at the endosome is required to maintain synaptic transmission at the NMJ. Analysis of the level of ubiquitinated proteins revealed a significant increase in ubiquitin conjugates specifically in the synaptosomal fraction of the *Hgs* ^*tn/tn*^ mice as compared to controls. While the numbers of myelinated and unmyelinated peripheral nerves in the *Hgs* ^*tn/tn*^ mice were similar to the numbers observed in wild type mice, the reduced HGS expression resulted in an increase in myelin thickness and dysmyelination of the sciatic nerve axons in the *Hgs* ^*tn/tn*^ mice. These results demonstrate that HGS is required to support both the myelination of peripheral nerves and synaptic transmission at the NMJ.

HGS regulates the sorting and trafficking of ubiquitinated, internalized receptors on the endosome [42,65,66]. Our finding that the M89V mutation in the VHS domain had variable effects on HGS expression in the different non-neuronal tissues examined suggests that HGS stability is dictated, at least in part, by its interaction with specific binding partners [54]. Alternatively, a variable demand for the ESCRT pathway may also be responsible for the differential effects of the M89V mutation on HGS expression in the various *Hgs* ^*tn/tn*^ tissues.

There are several similarities between the myelination defects detected in the *Hgs* ^*tn/tn*^ mice and those observed in the brain-derived neurotrophic factor (BDNF)-overexpressing mice [67]. Although we found no evidence for altered *BDNF* levels in the *Hgs* ^*tn/tn*^ mice, there was a significant reduction in TrkB in the sciatic nerves of *Hgs* ^*tn/tn*^ mice as compared to controls. However, there was no difference in the levels of *TrkB* mRNA detected in the *Hgs* ^*tn/tn*^ mice and controls, suggesting that HGS promotes TrkB stability by facilitating recycling to the plasma membrane and preventing trafficking to the lysosome. This conclusion is consistent with previous reports that HGS can regulate the recycling of TrkB.FL in neuronal culture [55]. It is possible that the limited effects of loss of HGS on TrkB expression may reflect a partial loss of HGS in the Schwann cells of the *Hgs* ^*tn/tn*^ mice. Our finding that STAM1 expression is not altered in *Hgs* ^*tn/tn*^ sciatic nerve extracts, but is reduced in spinal cord and brain extracts, may indicate that STAM1 associates with other proteins in Schwann cells to stabilize its expression or that the level of HGS expression was not sufficiently reduced in Schwann cells to destabilize STAM1. In addition, since HGS interacts with the NF2 gene MERLIN in Schwann cells [68,69], loss of this complex may contribute to the changes observed in the levels of TrkB. Since the observed changes in myelin thickness are very subtle, they are unlikely to be a major contributor to the neurological deficits observed in the *tn* mice.

Although the *Hgs* ^*tn/tn*^ mice represent the first report linking ESCRT-0 to neuromuscular disease, mutations in the ESCRT-III component CHMP2B have been linked to amyotrophic lateral sclerosis [27,28], and patients with CHMP2B mutations exhibit phenotypes consistent with lower motor neuron disease [28]. The *Hgs* ^*tn/tn*^ mice also show signs of dysfunction of the lower motor neurons, including motor incoordination, muscle weakness, muscle atrophy, hypokinesis, and reduced synaptic transmission at the NMJ. These deficits are not associated with loss of motor neuron cell bodies or axons in the *Hgs* ^*tn/tn*^ mice, or with denervation of the NMJ. In immortalized cell lines, expression of mutant CHMP2B resulted in the formation of dysmorphic endosomes [7], increased levels of autophagy markers, and the formation of ubiquitin-positive inclusions [28], suggesting that the motor neuron damage results from a block in autophagic clearance of ubiquitinated proteins. Although data from mammalian cell lines suggests that HGS is required for the maturation of autophagic vesicles, as depletion of HGS resulted in increased LC3 levels and decreased numbers of LC3/lysosome-associated membrane glycoprotein 1 positive structures in cell culture [44], loss of HGS resulted in reduced levels of the autophagy markers LC3 and p62 in the hippocampus of the *Hgs* ^*tn/tn*^ mice, indicating that loss of HGS does not appear to result in a blockade in autophagy in neurons.

Our studies demonstrate that HGS is required for synaptic transmission at the NMJ. The reductions in MEPC and EPC amplitudes and quantal content are consistent with reduced vesicle number and/or release at the NMJ of the *Hgs* ^*tn/tn*^ mice. These findings are also consistent with a previous report that showed that mutation of the neuronal Rab35 GTPase activating protein skywalker in *Drosophila* leads to an increase in the readily releasable pool of vesicles, enhanced neurotransmitter release and increased HGS-dependent protein sorting at the NMJ [25], suggesting that the ESCRT machinery is required for synaptic vesicle rejuvenation and removing defective synaptic vesicle proteins at the NMJ. The increased levels of ubiquitinated synaptic proteins detected in the *Hgs* ^*tn/tn*^ mice suggests that an ESCRT-dependent mechanism for clearing ubiquitinated proteins at synapses is also important to maintaining synaptic transmission in mammals. Alternatively, the increase in ubiquitinated conjugates may be an indirect effect caused by disruption of the endosomal sorting pathway.

HGS is known to interact with synaptic vesicle proteins and could therefore directly affect vesicle trafficking [52,56]. For example, by binding to SNAP25 [23,51], HGS can displace vesicle-associated membrane protein 2 and inhibit SNARE complex formation, thereby blocking neurotransmitter secretion and endosomal fusion [23,70]. While the presence of enlarged vesicles would be the predicted effect of HGS loss, we did not detect an increase in the size of the endosomes at motor neuron axon terminals in the *Hgs* ^*tn/tn*^ mice, but instead found an increase in the number of endosomes and MVBs at the NMJs of the *Hgs* ^*tn/tn*^ mice. Previous reports from a proteomic screen identified mammalian uncoordinated-18, a component of the synaptic vesicle fusion protein complex, as a protein that could interact with the ubiquitin-interacting motif of HGS [71]. HGS has also been shown to inhibit the fusion of vesicles with the early endosome [24]. Together, these results provide evidence that HGS interacts with components of the synaptic vesicle release machinery and plays a direct role in synaptic transmission.

Given that loss of HGS results in a predominantly neurological phenotype in the *Hgs* ^*tn/tn*^ mice, it is surprising that a neuronal-specific deletion of HGS [43] leads to such a mild phenotype compared to what we observed in our studies. Whereas *Hgs* ^*tn/tn*^ mice die by 5 weeks of age and present with ataxia, decreased muscle size, and reduced strength, the neuronal-specific deletion of HGS did not affect gait or grip strength, and there was no reported effect of HGS loss on viability [43]. However, it is possible that the conditional knockout allele used by Tamai et al. [43], which utilized a rat synapsin Cre driver [72], may not have led to a reduction of HGS in the motor neurons, or that the level of HGS was not reduced as much as what is observed in the *Hgs* ^*tn/tn*^ mice, thereby resulting in a milder phenotype.

Identification of the *tn* mutation in HGS has provided a new model for investigating the role of the ESCRT pathway in the nervous system. Our identification of motor and sensory phenotypes in both the *Hgs* ^*tn/+*^ and *Hgs* ^*KO/+*^ mice demonstrates that loss of HGS results in an autosomal dominant inheritance pattern similar to that observed in human cases of amyotrophic lateral sclerosis and Charcot-Marie-Tooth disease [27,28,35]. Hereditary neuralgic amyotrophy, an autosomal dominant form of recurrent focal neuropathy, is the only neurological disorder that has been mapped near *HGS* on chromosome 17q25 [73]. Mutations in Septin 9 have been found in many, but not all, cases of hereditary neuralgic amyotrophy, making *HGS* a good candidate gene for other cases of this disease. With our demonstration that HGS is required for motor neuron function, it will now be possible to determine if HGS is involved in other neurological disorders in humans.

## Materials and Methods

### Mice

The *tn* mutation spontaneously arose on the C3H/HeJ inbred mouse strain at The Jackson Laboratory in 1959. We generated a congenic line of B6.C3*Hgs* ^*tn*^ mice by backcrossing the *tn* mutation onto the C57BL/6J background for at least 12 generations. The *tn* mice were maintained by intercrossing *tn* sibling heterozygotes (*Hgs* ^*tn/+*^). *Hgs* knockout heterozygotes (*Hgs* ^*tm1Sor/+*^, stock number 003539) were obtained from The Jackson Laboratory, and sibling pairs were intercrossed to maintain the strain. Research was conducted using equal numbers of male and female mice. Wild type littermates or age-matched C57BL/6J mice (*Hgs* ^*+/+*^) were used as controls.

All mice were maintained in our breeding colony at the University of Alabama at Birmingham, which is fully accredited by the Association for Assessment and Accreditation of Laboratory Animal Care International. All research was approved by the UAB IACUC committee and complied with the United States Animal Welfare Act and other federal statutes and regulations relating to animals and experiments involving animals, and adhered to principles stated in the Guide for the Care and Use of Laboratory Animals, United States National Research Council.

### Genetic mapping

The *tn* mutation was previously mapped to distal chromosome 11 [74]. Small nucleotide polymorphism (SNP) analysis localized the *tn* mutation to a 2.8 Mb region on the distal end of chromosome 11 flanked by rs27043138 and rs3675603.

### RNA transcriptome analysis

Total RNA was isolated from brain lysates of 4-week-old *Hgs* ^tn/tn^ and *Hgs* ^+/+^ mice using RNA-STAT60 (Tel-Test, Friendswood, TX) and subsequently purified using an RNeasy Mini Kit (Qiagen Sciences, Valencia, CA). RNA-seq experiments were carried out at the Hudson Alpha Genome Services Laboratory (Huntsville, Alabama). Two μg of total RNA underwent quality control (Bioanalyzer; all RIN values > 9.5), and was prepared for directional RNA sequencing at Hudson Alpha using NEBNext reagents (New England Biolabs) according to manufacturer’s recommendations with minor modifications (including the use of custom library adapters and indexes). RNA libraries were quantified with the Kapa Library Quant Kit (Kapa Biosystems), and underwent sequencing (50 bp paired-end directional reads; ~70M reads/sample) on an Illumina sequencing platform (HiSeq2000).

### DNA sequencing

Exon 4 of *Hgs* was amplified from genomic DNA of *Hgs* ^tn/tn^ and *Hgs* ^+/+^ mice. The PCR products were sequenced by The Genomics Core Facility of the Heflin Center for Genomic Science (Birmingham, AL).

### Quantitative PCR

Total RNA was isolated from specified tissues using RNA-STAT60 and reverse transcribed using the Superscript VILO cDNA synthesis kit (Life Technologies). Individual gene assays were purchased from Applied Biosystems for each of the RNAs analyzed. ΔΔCt values were generated using *Hgs* (Mm00468635_m1), *Stam* (Mm00488457_m1), *AChRα* (Mm00431629_m1), *AChRβ* (Mm00680412_m1), *AChRδ* (Mm00445545_m1), *AChrδ* (Mm00437419_m1), *AChRε* (Mm00437411_m1), *Bdnf* (Mm04230607_s1) and TrkB Mm00435422_m1). Taqman gene assays with *18S* (Mm03928990_g1) and *β-actin* (Mm00607939_s1) served as internal standards. qPCR results are shown as the average of three different amplifications of cDNAs that were generated from at least three different mice. Unpaired Student’s t tests were conducted on ΔΔCt values from each genotype to determine their significance.

### Isolation of proteins

Mice of appropriate age and genotype were asphyxiated with CO_2_ or deeply anesthetized via isoflurane prior to rapid decapitation. Tissues were removed and homogenized in a modified RIPA buffer containing 50 mM Tris, pH 7.5, 150 mM NaCl, 5 mM MgCl_2_, 0.5 mM EGTA, 1 mM EDTA, 0.5% SDS, 1% Triton X-100, and 1% sodium deoxycholate. Complete protease inhibitors (Roche, Indianapolis, IN), phosphatase inhibitor cocktail I (Sigma Aldrich, St. Louis, MO), and 50 μM PR-619 (Life Sensors, Malvern, PA) were added to the homogenization buffer. Samples were homogenized in 1X Laemmli buffer, sonicated, and boiled. After homogenization, tissues were centrifuged at 17,000 x g for 10 min at 4°C, and supernatants were removed and immediately frozen at—80°C. Protein concentrations were determined by using the bicinchoninic acid (BCA) protein assay kit from Pierce (Rockford, IL).

### Immunoblot analysis

Proteins were resolved on either 10% Tris-glycine gels or 4–20% Novex Tris-glycine gels (Life Technologies) and transferred onto nitrocellulose or PVDF membranes. Immunoblots were probed for HGS, STAM1, TSG101, EPS15, EGFR (Santa Cruz, Dallas, TX), TrkA and TrkB (Millipore, Billerica, MA), CHMP2B (Abcam, Cambridge, MA), ERBB2, p62, LC3 (4108) and cleaved caspase 3 (Cell Signaling, Danvers, MA), mylein basic protein (Biolegend, Dedham, MA), GFAP (Dako, Carpinteria, CA) and ubiquitin (UAB Hybridoma Facility, Birmingham, AL). β-tubulin and β-actin (Developmental Hybridoma Bank, Iowa City, IA) were used for loading controls. Primary antibodies were diluted in 1X phosphate buffered saline containing 0.1% NP-40 and either 2% BSA or 1% non-fat dry milk, and proteins were detected by using an anti-mouse or anti-rabbit HRP-conjugated secondary antibody (Southern Biotechnology Associates, Birmingham, AL) and SuperSignal West Pico Chemiluminescent Substrate (Thermo Scientific, Rockford, IL).

### Quantitation of immunoblots

Blots were scanned using a Hewlett-Packard Scanjet 3970 (Palo Alto, CA) and quantitated using ImageJ (NIH, Bethesda, MD) or UN-SCAN-IT software (Orem, Utah). Each value represents the mean and standard error from at least two blots using at least three different animals per genotype. Unpaired t-tests, corrected for multiple comparisons using the Holm- Šídák method, were utilized to determine significant effects between genotypes. One-way ANOVAs with the Geisser-Greenhouse correction were used to analyze the developmental expression patterns of HGS.

### Body and muscle wet weight analysis

Gastrocnemius muscles were collected from 4-week-old *Hgs* ^+/+^ and *Hgs* ^tn/tn^ mice. Muscle and body weights were determined for at least six animals per genotype, and values are reported as the average muscle or body mass ± SE.

### Hippocampal immunostaining

Brain sections were prepared and stained as previously described [75].

### Sciatic nerve immunostaining and confocal microscopy

Sciatic nerves were dissected from 4-week-old wild type mice and immediately submersed in ice cold 4% PFA for 1 h. Sciatic nerves were cryopreserved in PBS containing 30% sucrose overnight at 4°C. Nerves were embedded in OCT (Tissue-Tek; Sakura Finetek USA) and 20 μM sections were cut for analysis. Sections were blocked in PBS containing 10% normal goat serum for 1 h at room temperature. Primary antibodies (HGS, S100β and Neurofilament) were diluted 1:200 in PBS containing 10% normal goat serum and incubated with sections overnight at 4°C. Sections were washed two times with PBS at room temperature and then incubated with secondary antibodies labeled with Alexa Fluor 488 or 568 dye (Invitrogen). Confocal imaging was performed on a Nikon C2 laser scanning confocal microscope using the NIS element advance imaging software package version 4.2 (Nikon, Melville, NY). Images were captured at 40x using a 0.3 μM z-step.

#### NMJ immunostaining and confocal imaging

Whole mount immunostaining of tibialis anterior (TA) muscles was performed as described [59]. Briefly, TA muscles were dissected and immersed in ice-cold 2% PFA in PBS for 1 h. Muscles were then teased into bundles and transferred to 1% PFA in PBST (PBS + 1% Triton) overnight at 4°C with constant rocking. Endplate size was determined by tracing the circumference of the α-bungarotoxin-positive post-synaptic AChR cluster and computing area using ImageJ software. Quantitation of axonal sprouts and swellings were performed using ImageJ software. Over 150 synapses from *Hgs* ^+/+^ and *Hgs* ^tn/tn^ mice were quantified.

### Behavioral analysis

Motor and sensory performance was assayed at 3 to 4 weeks of age with *Hgs* ^+/+^, *Hgs* ^tn/tn^, *Hgs* ^tn/+^, and *Hgs* ^KO/+^ mice (n ≥ 4). Before each behavioral assay, animals were habituated to the testing room for 30 min. Unpaired Student’s t-tests were performed on open field, grip strength and von Frey data. Two-way ANOVA was utilized to determine significance between genotypes on rotarod and elevated beam assays. Unpaired Student’s t-tests were utilized to determine the significance of each trial.

### Open field

Animals were handled at least three days prior to open field testing. Locomotor activity was measured in an open-field arena (43.2 cm x 43.2 cm x 30.5 cm) for 15 min by an automatic video tracking system (Med Associated, St. Albans, VT). The first 5 min were not analyzed to account for habituation to the open field chamber.

### Rotarod

Motor coordination and balance were tested by placing mice on an accelerating rotarod (ENV-575, Med Associates) and recording latency to fall. The rotarod initially started rotating at 3.5 rpm and accelerated to 35 rpm over a 5 min period. Each mouse performed 3 trials separated by 1 h.

### Elevated beam

Motor coordination and proprioception were tested by the elevated beam assay as previously described [76]. Four trials were performed with each trial consisting of 3 repetitions of the assay.

### Grip strength

The San Diego Instrument Grip Strength System (San Diego, CA) was used to assay mouse grip strength. The maximum amount of force generated from forelimbs was recorded. Each trial consisted of 12 repetitions of the assay with the two highest and two lowest data points dropped from final analysis.

### Von Frey analysis

Animals were habituated to an open gridded floor chamber for 5 min. A series of 10 von Frey fibers varying from 0.4 g to 60 g of force (Ugo Basile, Comerio, Italy) was applied from below the wire mesh chamber in ascending order beginning with the smallest fiber. The fiber was applied to the central region of the plantar surface to avoid the foot pads. The hind paw withdrawal threshold was determined by Dixon’s formula.

### NMJ transmission electron microscopy

TA muscle tissues were fixed in 4% paraformaldehyde in 0.1 M sodium cacodylate buffer for 1 h. Fixed muscle fibers were stained with α-bungarotoxin and dissected to isolate regions of muscles containing NMJs. Muscles were washed in 0.1 M sodium cacodylate buffer and then post-fixed in 1% osmium tetroxide for 1 h in the dark. After rinsing with sodium cacodylate, the samples were dehydrated in increasing concentrations of acetone (50% for 10 min, 75% for 10 min, 90% for 10 min, 95% for 10 min, 100% for 4 x 10 min). Muscles were transitioned to epoxy resin (Electron Microscopy Sciences, Hatfield, IL) by rotating the samples overnight in a 50:50 solution of epoxy:acetone. The samples were then immersed in fresh 100% resin several times throughout the next day and baked at 65°C overnight. Ultra-thin cross-sections were collected using a Leica EM-UC6 ultramicrosome (Buffalo Grove, IL) and stained for contrast with uranyl acetate and lead citrate. Samples were viewed using an FEI Tecnai T-12 electron microscope (Delmont, PA) with a Hamamatsu digital camera (Bridgewater, N.J).

### Quantitation of motor neuron number

For quantitation of motor neuron numbers, transverse frozen sections of lumbar spinal cords (L4 /L5) from 7-week-old wt and *axJ* mice were prepared and stained with cresyl violet as previously described [77].

### Morphometric analysis of the NMJ

Ultra-thin sections of muscle tissues were scanned for the presence of NMJs. Micrographs were taken at 4400-6500x magnification. A total of 14 synapses were analyzed from *Hgs* ^+/+^ mice (n = 3), and 27 synapses from *Hgs* ^tn/tn^ mice (n = 3) were quantified. Endosomes were defined as single membrane intracellular vesicles that were at least 100 nm in feret diameter, and MVBs were defined as endosomes with at least 2 ILVs within the lumen. Endosomes and MVBs were quantified and normalized to the size of the synapse.

### Transmission electron microscopy of sciatic nerves

Sciatic nerves were excised from 4-week-old *Hgs* ^+/+^ and *Hgs* ^tn/tn^ mice and immersed in 6% gluteraldehyde/2% paraformaldehyde in 0.1 M sodium cacodylate buffer for at least 1.5 h at room temperature. Samples were processed as described above for NMJ analysis.

### Morphometric analysis of sciatic nerves

Photomicrographs for morphometric analysis were obtained by systematically covering adjacent but non-overlapping fields to measure axon and myelin diameter, myelin thickness, and myelin defects. The feret diameter was calculated to determine axon and myelin caliper. The G-ratio was also calculated by dividing the axon diameter by the myelin diameter. Quantitation was performed using ImageJ software. A total of 595 myelinated axons from *Hgs* ^+/+^ mice (n = 3) and 574 axons from *Hgs* ^tn/tn^ mice (n = 3) were quantified. Micrographs taken at 240x magnification were utilized to calculate axon density of myelinated fibers, while micrographs taken at 1100x were used to quantitate the density of unmyelinated fibers.

### Electrophysiology

All experiments were carried out *in vitro* at room temperature on the sciatic nerve and TA muscles from 3- to 4-week-old mice. For dissection, mice were deeply anesthetized with isofluorane. The TA muscle was dissected free from the extensor digitorum longus (EDL) muscle, partially bisected, and folded apart to flatten the muscle before pinning it down. Because the sciatic nerve innervates both EDL and TA muscles, we transected the EDL and posterior portion of the TA muscle to eliminate excessive muscle contraction. The TA muscle was perfused with Tyrode's solution (137 mM NaCl, 2.8 mM KCl, 1.8 mM CaCl_2_, 1.1 mM MgCl_2_, 11.9 mM NaHCO_3_, 0.33 mM NaH_2_PO_4_, and 11.2 mM dextrose, pH 7.4, when bubbled with a mixture of 95% O_2_ and 5% CO_2_) at 30°C. Intracellular potentials and currents were measured using an Axoclamp-2A amplifier system and glass microelectrodes filled with 3 M KCl (resistance, 10–15 MΩ). Using the two-microelectrode voltage-clamp system, synaptic currents, including miniature endplate currents (MEPCs) and endplate currents (EPCs), were obtained at -60 mV holding potential. The currents were digitized at 50 μs per point and were stored, captured, and analyzed using pClamp 9.0 software (Molecular Devices). EPCs were elicited by stimulating the corresponding sciatic nerve with rectangular pulses of 0.05 ms duration. During single shock stimulation, the nerve was stimulated at 0.2 Hz. The amplitude decay and time to peak of the EPCs and MEPCs were captured and subsequently analyzed with pClamp 9.2 software and the Mini Analysis Program (Synaptosoft). Quantal content was determined by dividing MEPC amplitude with EPC amplitude from the same endplate [78–80].

### Synaptosomal fractionation

Synaptosomes were prepared from 4-week-old cerebral cortices of wild type and *tn* mice as described [81].
